# One size does not fit all in evaluating model selection scores for image classification

**DOI:** 10.1038/s41598-024-81752-w

**Published:** 2024-12-04

**Authors:** Nermeen Abou Baker, Uwe Handmann

**Affiliations:** Computer Science Department, Ruhr West University of Applied Sciences, Bottrop, Germany

**Keywords:** Image classification, Model ranking, Model selection, Transfer learning, Transferability estimation, Computer science, Information technology, Statistics

## Abstract

Selecting pretrained models for image classification often involves computationally intensive finetuning. This study addresses a research gap in the standardized evaluation of transferability scores, which could simplify model selection by ranking pretrained models without exhaustive finetuning. The motivation is to reduce the computational burden of model selection through a consistent approach that guides practitioners in balancing accuracy and efficiency across tasks. This study evaluates 14 transferability scores on 11 benchmark datasets. It includes both Convolutional Neural Network (CNN) and Vision Transformer (ViT) models and ensures consistency in experimental conditions to counter the variability in previous research. Key findings reveal significant variability in score effectiveness based on dataset characteristics (e.g., fine-grained versus coarse-grained classes) and model architectures. ViT models generally show superior transferability, especially for fine-grained datasets. While no single score is best in all cases, some scores excel in specific contexts. In addition to predictive accuracy, the study also evaluates computational efficiency and identifies scores that are suitable for resource-constrained scenarios. This research provides insights for selecting appropriate transferability scores to optimize model selection strategies to facilitate efficient deployment in practice.

## Introduction

Deep learning has outperformed many supervised tasks due to large benchmark datasets, finetuned pretrained models (PTMs), and specialized computing devices like GPUs. Transfer learning is a technique that aims to reuse knowledge learned from a source task to improve performance on a target task. It has emerged as a de facto standard in adapting learning knowledge across various applications. Particularly in the vision and language tasks, transfer learning from benchmark datasets and suitable PTMs has achieved tremendous success, and it provides a remarkable improvement especially when limited annotated datasets are available. These models provide a very good starting point for finetuning target datasets. The collection of PTMs, which vary in architecture and size, is commonly referred to as a model zoo, model pool, model hub, or checkpoints. These models require less training time and converge faster than training from scratch. In supervised image classification, the most common practice is using the weights trained on ImageNet and subsequently finetuning them on new target datasets. These checkpoints are publicly available in many repositories, such as Tensorflow^1^, Pytorch^2^, and Hugging Face transformers^3^.

In transfer learning, the initial challenge is selecting the most suitable pretrained model for the specific task. This problem is not trivial and can lead to negative transfer. However, not all PTMs perform equally well on all target datasets. As the number of PTMs increases, including CNNs or ViT backbones, the question arises:

**Given the variety of pretrained models, how can the most appropriate pretrained model be quickly and accurately selected for a given target dataset?**

Finetuning a pretrained model on one target dataset can take several GPU hours, then selecting the best-pretrained model and finetuning various hyperparameters such as learning rates and optimizers can take GPU days or GPU weeks, which is beyond the reach of many average machine learning practitioners. This method is known as brute-force finetuning. It is a greedy approach because it is computationally expensive and time-consuming, especially for a large number of PTMs.

Ranking PTMs based on classification accuracy has been a critical issue in many previous studies. This ranking should be computationally efficient and correlate well with the ground truths of the target dataset. Hence, this problem motivates the need for an efficient model selection method. Alternative methods to brute-force try to rank the PTMs without finetuning them. For instance, some methods suggest that finetuning performance is related to the distance between tasks, such as Taskonomy, which defines this distance a posteriori by analyzing finetuning accuracy during transfer, while others use the Earth Mover Distance (EMD) between source and target features to predict domain similarity^4^. However, these measures produce different results when transferring from one task to another than when transferring in the opposite direction. Task2Vec builds task representations using the Fisher matrix of trained weights and measures task similarity using the cosine distance between these representations, which helps predict transferability^5^. Although task relationships can be modeled by measuring divergence between their generating distributions, this approach becomes difficult when the target dataset is small or labels are limited. On the other hand, Optimal Transport (OT) assumes that the source and target datasets have similar geometric structures and are closely aligned in distance. However, as previously discussed, this distance metric lacks accuracy. Additionally, implementing OT requires access to the source training set, which is computationally infeasible for large datasets^6^.

To address these problems, a new trend in transferability metrics is continuously emerging to predict the ranking of these PTMs, a process known as model selection or transferability estimation. The main idea of these metrics is to apply the source model to the target dataset to compute predictions or embeddings, and then evaluate how compatible they are with the target labels. Thus, they serve as proxies for how well the source model transfers to the target task. However, small changes in the experimental setup can greatly impact results, suggesting that the claimed superiority of these scores may apply only to specific conditions. To ensure consistency across various metrics, it is essential to standardize the experimental setup by unifying factors such as tested datasets and PTMs. This study re-implements all metrics to ensure fair comparisons. It evaluates 14 metrics, on 11 benchmark classification datasets using a model pool containing 11 pretrained CNN and 10 pretrained ViT models. The transferability metrics are: The ImageNet weights, Negative Conditional Entropy (NCE)^7^, H-score^8^, Log Expected Empirical Prediction (LEEP)^9^, $\mathscr {N}$LEEP^10^, Logarithm of Maximum Evidence (LogME)^11^, Transferability Rate (TransRate)^12^, Gaussian Bhattacharyya Coefficient (GBC)^13^, Label-Feature Correlation (LFC)^14^, Probably Approximately Correct Transfer (PACTran)^15^, Self-challenging Fisher Discriminant Analysis (SFDA)^16^, Neural Collapse Transferability Index (NCTI)^17^, Efficient Multi-task Model Selector (EMMS)^18^, and Energy-based Transfer (Etran)^19^. Additional details about the tested transferability metrics will be provided later.

In summary, the main contributions of this paper are as follows:

- Comprehensive evaluation of transferability scores: The research performs a comprehensive empirical evaluation of 14 different transferability scores on 11 benchmark image classification datasets. This comprehensive analysis uses a diverse model pool consisting of 11 pretrained CNN models and 10 ViT models. By examining a wide range of transferability metrics under consistent conditions, the study provides clear, comparative insights into the effectiveness of these scores in predicting model transferability.
- Standardization of the experimental design: Introduces a standardized experimental design that addresses a key limitation of previous research. Previous studies have shown significant variability in score performance across different experimental conditions, which can lead to misleading conclusions about the appropriateness of transferability scores for specific tasks. This work ensures consistency by controlling for critical factors such as dataset characteristics, model architectures, evaluation metrics, and computational efficiency, allowing for more reliable comparisons and insights.
- Analysis of computational efficiency: This study goes beyond simply evaluating classification accuracy to examine the computational efficiency of each transferability score. By incorporating efficiency metrics alongside performance measures, the research identifies scores that are suitable for resource-constrained environments. This analysis provides valuable guidance to practitioners who must balance model performance with available computational resources.
- Guidance for practitioners: Considering the trade-offs between accuracy, computational efficiency, and dataset properties, the study provides comprehensive guidelines to assist practitioners in selecting appropriate transferability scores for various image classification scenarios. While no single score emerges as universally superior, the research highlights the strengths of specific scores in particular contexts, enabling informed decision-making.

By addressing the limitations of previous work and providing a thorough, standardized evaluation, this study significantly advances the understanding and application of transferability scores in image classification tasks. The gained insights are of significant value to researchers and practitioners seeking robust, efficient model selection strategies to support transfer learning in diverse real-world applications.

## Review of related work

The field of model selection for image classification has advanced significantly, with the introduction of various transferability scores aimed at optimizing model performance across different datasets. These advances indicate the growing importance of efficient and adaptable methods for selecting models that are best suited for specific tasks and domains. This literature review is divided into two sections:

- Section “Introduction” reviews previous research closely related to this work, focusing on benchmarking and model zoo development, improvements in CNN model selection, and evaluation of transferability scores in domains such as medical imaging, radio frequency, and natural language processing (NLP). This section also discusses large-scale evaluations and the practical challenges of long-tail tasks, as well as efforts to bridge theoretical and practical aspects of CNN transferability.
- Section “Review of related work” addresses further studies with potential applications in model selection. This section categorizes relevant research into three areas: Efficient model selection for specialized applications, representation and similarity-based methods, and feature separability and evolution-based approaches. While these studies provide valuable insights, they have limitations that limit their direct applicability to this work; however, they provide promising directions for future research.

This structured review identifies the basic approaches and remaining gaps in transferability score applications, laying the groundwork for this study’s focus on improving model selection frameworks.

### Section 1: Evaluation of transferability scores and model selection approaches

This section examines core themes across the existing literature, including the following related work:

#### Empirical evaluation of pretrained CNN selection

A foundational study in this area empirically evaluated model selection using metrics such as accuracy, accuracy density, training time, and model size. This evaluation was based on a pool of 11 pretrained CNN models tested on five datasets. The study used a brute-force approach by averaging the performance results to provide practitioners with guidelines for selecting the most appropriate pretrained model according to specific problem requirements. Although this approach provided practical insights into model selection strategies and introduced a trade-off of decision factors, it did not hold for fine-grained datasets and has generalization problems^20^.

#### Advances in CNN model selection

Recent research has explored how the reasoning capabilities of Large Language Models (LLMs) such as GPT4 can optimize model selection by analyzing descriptions of specific use cases and autonomously selecting models based on task requirements. This novel method differs from brute-force approaches in that it eliminates the need for manual model testing across datasets. In this study, GPT4 was tasked with selecting PTMs for ObjectNet, a real-world image classification dataset containing 100 evaluation images. This LLM-assisted selection reduced the need for extensive manual evaluation, suggesting the potential for real-world efficiency gains. However, this method was only tested on a single dataset, raising concerns about its generalizability to other domains^21^.

#### Evaluation of transferability scores in medical imaging

In the field of medical image classification, another study evaluated the performance of seven transferability scores, namely H-score, NCE, LEEP, $\mathscr {N}$LEEP, LogME, regularized H-score, and GBC, in three medical applications using ten pretrained CNN models. Despite extensive analysis, the study found that the transferability scores were unstable in both in-distribution (IND) and out-of-distribution (OOD) settings. The lack of consistent performance led the authors to not recommend these scores for medical applications. The authors identified possible reasons for this instability due to the domain shift between the source and target datasets^22^.

#### Evaluation of transferability scores in radio frequency and NLP

In contrast to the previous study, research in the radio frequency domain has shown that transfer learning can be effectively guided by transferability scores. The study evaluated 23 types of signals using basic CNN architectures. The study finds a strong correlation between these transferability scores and post-transfer performance, suggesting their utility in predicting model accuracy without extensive retraining. However, the study is limited to using LEEP and LogME for transferability scores, ensuring the need to include robust scores^23^.

Similarly, in NLP, LogME was used to rank pretrained language models on ten datasets involving classification and structure prediction. The study reported a positive correlation between LogME rankings and final model performance in 94% of the setups tested, providing robust evidence of score reliability. The study identified a limitation in predictive accuracy after full model finetuning, which was attributed to a mismatch between frozen and finetuned representations. In contrast, initial experiments with alternative scores, such as LEEP, showed low correlations, indicating the need for further research to find scores that are better suited for NLP tasks^24^.

#### Large-scale evaluations and challenges in long-tail tasks

To further advance the field, a large-scale study conducted 715, 000 of experiments to evaluate several transferability scores, including five transferability scores, LEEP, $\mathscr {N}$LEEP, LogME, GBC, and OTCE, on nine benchmark datasets, such as Cifar10, Cifar100, Oxford Pets, and Stanford Dogs, using 19 pretrained CNN models. The results showed that even small variations in experimental setups can lead to different conclusions about the effectiveness of transferability scores. Nevertheless, LogME emerged as the best candidate for selecting source models, $\mathscr {N}$LEEP for selecting model architectures, and GBC for identifying the optimal target dataset. However, the study found that no single score consistently outperformed others across all scenarios, indicating the complexity of evaluating transferability and the need for standardized evaluation frameworks. The study recommended future research to explore more robust approaches to transferability assessment^25^.

In addition, another investigation focused on the evaluation of LFC, LogME, and Pairwise Annotation Representation Comparison (PARC) using over 400 models finetuned on 40 downstream datasets. This work focused on the challenges posed by long-tailed tasks where the data distribution is highly unbalanced. The study concluded that no single model performs optimally across all tasks, and recommended the need for tailored model selection strategies in these scenarios^26^.

#### Bridging theory and practice in CNN transferability assessment

A recent review examined all available transferability scores and categorized them into source-independent and source-dependent estimates. While the review described the concepts, it remained abstract and lacked empirical validation. In contrast, This current work aims to fill this gap by empirically evaluating all available transferability scores within a standardized execution setup, using popular computer vision datasets to assess their performance results^27^.

Table 1 summarizes key contributions from current studies in this field to provide a comprehensive overview of recent advancements in model selection evaluation.

**Table 1:** Summary of previous key contributions in model selection evaluation.

Study	Year	Key contributions	Techniques used	Target applications	Limitations
Abou Baker et al.^20^	2022	Model selection, transferability scores (accuracy, training time, model size), 11 pretrained CNNs, five datasets	Brute-force, averaging performance	Image classification	Generalization to multiple dataset characteristics
Wong et al.^23^	2022	Transferability score correlation, 23 signal types, basic CNN architectures	LEEP, LogME	Radio frequency signal classification	Limited to 2 scores
Bassignana et al.^24^	2022	LogME for ranking PTMs, high correlation in $94\%$ of cases	LogME, LEEP	NLP, classification, structure prediction	Limited to 2 scores
Agostinelli et al.^25^	2022	Evaluated 5 scores, 715, 000 experiments, 9 datasets, LogME best for model selection	LEEP, $\mathscr {N}$LEEP, LogME, GBC, OTCE	Image classification, model selection	Variability in results, no consistent best score, and no guidelines provided
Chaves et al.^22^	2023	Instability of transferability scores, 7 scores, 3 medical imaging tasks, 10 pretrained CNNs	H-score, NCE, LEEP, $\mathscr {N}$LEEP, LogME, regularized H-score, and GBC	Medical image classification	Inconsistent performance (IND/OOD)
Kannan et al.^21^	2023	GPT-4, task-specific model selection, ObjectNet dataset	GPT-4, large language models	ObjectNet classification dataset	Single dataset
Li et al.^26^	2023	Evaluation of LFC, LogME, PARC, 400 models, 40 datasets, long-tailed tasks	LFC, LogME, PARC	Long-tailed tasks, image classification	Limited to 3 scores
Ding et al.^27^	2024	Categorized transferability scores, source-independent and dependent, lack of empirical validation	Transferability score categorization	Model selection	Theoretical study, no empirical validation

### Section 2: Further related studies

The advancement in transfer learning has demanded a robust framework for pretrained model selection, emphasizing methods that predict model compatibility with target tasks. The literature on transferability estimation is diverse, comprising three major categories:

#### Efficient model selection for specialized applications

Recent developments in PTMs selection provide efficient strategies for adapting models to specific tasks. These approaches aim to optimize model performance while minimizing computational cost and aim to optimize specialized datasets.

*B-Tuning* This paper presents an approach to model selection that avoids the need for exhaustive finetuning across all model combinations. It proposes LogME that estimates model compatibility by maximizing label evidence, achieving a 3700x speedup over traditional methods^28^. Paired with LogME, the Bayesian finetuning procedure B-Tuning further improves model performance by accommodating heterogeneous PTMs, making the framework adaptable to diverse datasets and tasks. While the approach demonstrates significant efficiency gains and broad applicability, it has limitations, notably its partial ability to capture complex model-dataset interactions and its lack of architectural or hardware-specific considerations.

*MELEP* is specifically designed for multi-label classification tasks in medical applications, such as ECG analysis^29^. It adapts the LEEP methodology to address the unique challenges of multi-label health tasks, which have been largely ignored in previous research. MELEP evaluates the compatibility of PTMs with multi-label ECG tasks through forward passes. When benchmarked against LEEP, MELEP demonstrates superior efficiency, especially in low-resource and unbalanced data settings, and is evaluated on 12-lead ECG datasets. While MELEP excels in multi-label contexts, its applicability is primarily limited to domains similar to ECG analysis, and it may not perform as effectively in single-label tasks or other medical domains significantly different from those tested.

#### Representation and similarity-based methods

Alternative techniques in model transferability prioritize efficient, accurate model selection by moving beyond exhaustive forward passes. These methods use different frameworks that improve cross-task compatibility assessment by addressing dependencies in feature and observation spaces, although challenges remain in scaling to larger, more complex datasets.

*Model SPIDER* uses a representation-based framework that tokenizes tasks and models into vector representations to assess compatibility^30^. Using a transformer module, the Model SPIDER computes similarity scores between PTMs and tasks, dynamically re-ranking models based on available computational resources. Unlike traditional forward-pass-dependent methods, this approach achieves efficient model selection without exhaustive forward passes, balancing accuracy and computational cost. When benchmarked against standard forward pass strategies, Model SPIDER demonstrates adaptability to different model hubs, including visual models and LLMs. While innovative in its tokenization approach, its effectiveness depends on the quality of the pretrained representations and may struggle with tasks that differ significantly from those used for pretraining.

*Duality diagram similarity (DDS)* uses a duality diagram framework that captures both individual observations and feature dimensions to measure model compatibility^31^. This method excels in task transfer learning, especially in vision-based tasks such as Taskonomy, Pascal VOC, and NYUv2, where accurate model ranking across different layers is critical. DDS differs from previous work by emphasizing feature and observation dependencies, which enhances its ability to predict transferability with high correlation. Compared to Taskonomy methods, DDS achieves a 10% improvement in ranking accuracy on the Taskonomy dataset. However, its computational complexity limits its scalability, especially for large models and datasets, limiting its application to more computationally feasible scenarios.

#### Feature separability and evolution-based approaches

Other transferability methods focus on feature separability and optimization dynamics to enhance model compatibility predictions, offering improved ranking accuracy across diverse datasets but facing constraints in hardware adaptability and theoretical generalization.

*KITE* is a kernel alignment-based method that measures model compatibility by assessing feature separability and similarity to random features^32^. It evaluates the degree to which pretrained features align with the requirements of the target task, using centered kernel alignment as the metric. This dual focus on feature separability and random feature similarity provides insights beyond simpler distance metrics, allowing for more reliable transferability predictions across visual tasks. KITE outperforms several methods in a large-scale benchmark that includes 8 sources and 6 target datasets, with strong correlations in Pearson and Kendall’s rank metrics. However, KITE does not consider hardware constraints or resource efficiency.

*LEAD* takes a differential equation-based approach in logit space, aligning transferability estimation with finetuning optimization objectives^33^. Unlike previous methods that focus on linear transformations in feature space, LEAD uses differential equations to model nonlinear optimization dynamics, which is particularly beneficial for tasks with high and low data availability. LEAD’s focus on finetuning objectives yields a 17% gain in rank correlation with transfer performance over baseline methods, positioning it as a highly accurate option for real-time transferability estimation. Its evaluation includes 24 supervised and self-supervised models across 10 downstream datasets covering a wide range of visual tasks. Despite its effectiveness, LEAD’s focus on logit-based modeling may not generalize well to non-logit architectures, and its assumptions in the theoretical framework may limit its adaptability.

### Research gap

Current research reveals critical limitations in transferability score stability and cross-domain applicability. Existing methods show inconsistent performance across different datasets, and no single metric or selection strategy has achieved optimal performance in all scenarios. This study addresses these limitations by introducing a standardized framework for evaluating transferability scores and conducting a comprehensive empirical analysis of widely used image classification datasets. This research provides practitioners with robust, efficient model selection tools that balance accuracy and computational efficiency while extending the current understanding of transferability metric behavior across domains and architectural selections.

## Overview of transferability metrics

This study evaluates various transferability scores, presented chronologically from 2019 to 2023.

### ImageNet weights

One of the earliest attempts to understand transferability estimation involved the brute-force method with extensive finetuning on classification datasets^34^. The study proves a strong correlation between transfer accuracy and ImageNet accuracy. However, this correlation does not hold for fine-grained datasets, where classes have high distinctions and granularity, especially if these labels are underrepresented in ImageNet. In addition, it is shown that better pretrained architecture can learn better features.

### Negative conditional entropy (NCE)

Negative Conditional Entropy (NCE)^7^ differs from previous methods by directly assessing the compatibility between the source and target label distributions. Using concepts from information theory, NCE measures transferability by minimizing the conditional entropy $H(Y|Z)$ between the label sequences of the source dataset $Y$ and the target dataset $Z$, assuming the datasets share identical input examples with varying labels. The conditional entropy $H(Y|Z)$ is defined as:

1 $$\begin{aligned} H(Y|Z) = -\sum _{z \in Z} P(z) \sum _{y \in Y} P(y|z) \log P(y|z) \end{aligned}$$

where: $P(y|z)$ is the probability of observing label $y$ given label $z$. $P(z)$ is the marginal probability of observing $z$.

This measure quantifies the uncertainty in predicting $Y$ given knowledge of $Z$, with lower conditional entropy indicating higher transferability, as it reflects a greater alignment between the source and target labels. The NCE value is simply the negative of this computed entropy:

$$\text {NCE} = -H(Y|Z)$$

This straightforward computation provides an efficient measure of alignment between label distributions across tasks, effectively quantifying transferability.

### H-score

H-score^8^ is based on the intuition that a model will transfer well to a target dataset when there are small differences between classes and low redundancy. Using principles from statistics and information theory, it measures feature performance by looking at the average log-loss when using input features to predict target labels. The H-score assumes both data come from the same domain. To achieve this, the H-score calculates class separation in the feature space by analyzing covariance matrices and class means, applying principles from Euclidean information geometry. However, the H-score treats the classification problem as a linear regression task and relies on the least squares solution, which may not work well when target labels and input are closely related.

H-score considers two factors: high inter-class variance (strong separation between classes) and low redundancy within features. It uses the following equation to assess feature transferability:

2 $$\begin{aligned} H(f) = \text {tr}\left( \text {cov}(f(X))^{-1} \cdot \text {cov}(E[P_{X|Y}[f(X)|Y]])\right) \end{aligned}$$

where $\text {tr}$ is the matrix trace, $\text {cov}(f(X))$ denotes the covariance of feature representations, $E[P_{X|Y}[f(X)|Y]]$ represents the expected feature values given each class $Y$.

This equation shows how the H-score uses statistical measures to capture the relationship between inter-class variance and redundancy. A higher H-score suggests better transfer potential, as it reflects greater separation between classes with minimal overlap.

In evaluating transferability, the H-score can be part of a ranking strategy for models, with models that achieve higher H-scores on a target task generally having better transfer potential.

### Log expected empirical prediction (LEEP)

LEEP^9^ is an information-theoretic method for assessing transferability between two classification tasks by analyzing the correlation between their label sequences. The method is designed to provide an upper bound on negative conditional entropy and has been shown to outperform this measure as a transfer metric empirically. Unlike NCE, LEEP does not require assumptions about the source and target data, except that they share the same input dimensionality. In LEEP, the source labels used in NCE are replaced with pseudo-labels generated by a pretrained source model.

The LEEP score is calculated by estimating the empirical conditional distribution from the joint distribution of the pseudo-source labels and the target labels. The process begins by using the source model to predict pseudo-labels for target data and then computing the log-likelihood between the predicted labels and the actual target labels. The underlying rationale is that if the predictions concentrate around specific target labels, adapting to the target dataset should be easier. However, this method is restricted to classification tasks due to its reliance on a Softmax output layer.

Moreover, LEEP evaluates how effectively a source-trained model can perform on a target dataset without additional training by estimating the log-likelihood of an Expected Empirical Predictor (EEP). The EEP acts as an intermediary classifier that integrates the source model’s outputs with empirical statistics from the target data. It is calculated as follows:

1. Expected Empirical Predictor (EEP): For an input $x$ from the target dataset, the source model $\theta$ outputs a probability distribution over source labels $Z$. The probability of predicting the target label $y$ for $x$ is defined as:
  $$P(y|x; \theta , D) = \sum _{z \in Z} P(y|z) \cdot \theta (x)_z$$
  where $\theta (x)_z$ is the probability assigned by the source model to label $z$, and $P(y|z)$ is the empirical conditional distribution between target and source labels.
2. Computing the LEEP Score: The LEEP score is the average log-likelihood across the target dataset $D$:
  3 $$\begin{aligned} \text {LEEP}(\theta , D) = \frac{1}{n} \sum _{i=1}^{n} \log \left( \sum _{z \in Z} \hat{P}(y_i|z) \cdot \theta (x_i)_z \right) \end{aligned}$$
  where $\hat{P}(y|z)$ is computed from the empirical data, $\theta (x_i)_z$ represents the source model predictions, and $n$ is the number of samples in $D$.

The LEEP score quantifies how well a source model’s predictions align with the target labels, making it a useful metric for assessing transferability. Higher LEEP scores indicate a stronger alignment, suggesting that the source model is more compatible with the target task, thereby aiding in the selection of models for finetuning or direct application.

However, there are limitations to consider. One concern is the risk of overfitting due to LEEP’s reliance on the empirical conditional distribution, which might not maximize the log-likelihood needed for the best target task performance. Additionally, the LEEP score’s computation over the target dataset can act more like a training error than a true generalization measure, potentially leading to overfitting if not properly accounted for. Despite these limitations, when overfitting is managed, the LEEP score remains a reasonable proxy for evaluating model performance on target tasks.

### $\mathscr {N}$lEEP

$\mathscr {N}$LEEP^10^ improves the LEEP score by implementing neural checkpoints, which enhance feature separability, addressing the challenges posed by diverse PTMs.

To overcome the limitations of LEEP, $\mathscr {N}$LEEP implements several key modifications.

- First, it replaces the output layer of checkpoints with a Gaussian Mixture Model (GMM) to provide a more robust density estimation of the pseudo-labels. The GMM is fitted to the target dataset, while LEEP’s softmax classifier is trained on the potentially mismatched source data, leading to improved performance in estimating posterior probabilities. The GMM is expressed as:
  $$P(s) = \sum _{v \in V} \pi _v \mathscr {N}(s | \mu _v, \Sigma _v),$$
  where $\pi _v$ are the mixture weights, and $\mathscr {N}(s | \mu _v, \Sigma _v)$ represents the Gaussian distributions characterized by the mean $\mu _v$ and covariance $\Sigma _v$ for each cluster $v$.
- Second, $\mathscr {N}$LEEP enhances the soft assignment method of the GMM by substituting pseudo-labels with clustering indices. This adjustment refines the calibration of cluster assignment probabilities, allowing for a more accurate reflection of the model’s capability to generalize to the target task.
- Third, dimensionality reduction is applied to the penultimate layer outputs via Principal Component Analysis (PCA) before fitting the GMM. This process simplifies the high-dimensional feature space, facilitating more efficient clustering and probability estimation. The $\mathscr {N}$LEEP ranking score is computed as follows:
  4 $$\begin{aligned} \mathscr {N}\text{ LEEP } (\theta ) = \frac{1}{n} \sum _{j=1}^{n} \log \sum _{v \in V} P(y_j | v) P(v | s_j), \end{aligned}$$
  where $P(y_j | v)$ is the probability of the target labels conditioned on GMM components, and $P(v | s_j)$ denotes the posterior probabilities. This ranking mechanism empowers practitioners to select the most promising checkpoints before the computationally intensive finetuning phase, particularly in domains with scarce labeled data.

Despite these advancements, $\mathscr {N}$LEEP still encounters challenges related to non-optimal log-likelihood and generalization. However, its ability to rank pretrained neural networks effectively signifies its potential impact on optimizing transfer learning outcomes.

### Logarithm of maximum evidence (LogME)

**Fig. 1:**
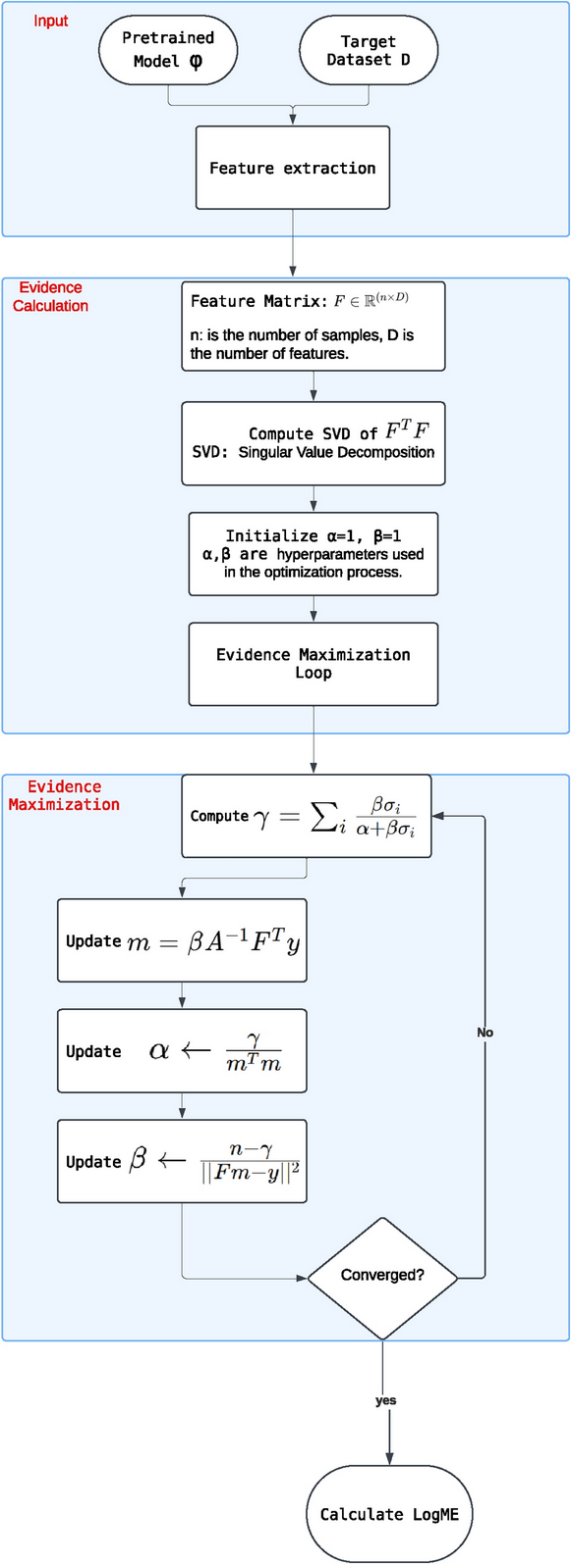
Calculating the LogME score.

LogME^11^ follows an approach similar to the H-score. Like the H-score, LogME uses the least squares objective function. However, instead of directly minimizing the Gaussian-based log-likelihood or square loss, LogME uses Bayesian averaging to avoid overfitting and improve generalization. To estimate transferability, LogME calculates the evidence, known as the maximum label marginalized likelihood, through a graphical model. LogME deals with each target label as a linear model with Gaussian noise. It then optimizes the parameters of the prior distribution to find the average maximum evidence. Furthermore, LogME computes the probability of the target labels conditioned on these embeddings, known as the target label evidence. Figure 1 visualizes the calculation process of the LogME score.

### Transferability rate (TransRate)

TransRate^12^ measures the transferability of PTMs as the mutual information between features extracted by the pretrained model and their corresponding labels. It addresses the challenge of estimating transferability without requiring access to target data for optimization. To achieve this efficiently, the coding rate is introduced as an alternative to entropy for estimating mutual information, which is closely related to rate distortion and entropy in information theory. TransRate proposes a computationally efficient method that measures mutual information between features from the source and target tasks, providing both upper and lower bounds for log-likelihood. This approach aids in effectively selecting PTMs and layers for transfer learning applications.

Mathematically, TransRate is defined as follows:

5 $$\begin{aligned} TrRTs \rightarrow T_t(g) = h(Z) - h(Z|Y) \approx H(Z_\Delta ) - H(Z_\Delta |Y) \end{aligned}$$

where $Z = g(X)$ represents the features obtained from the pretrained feature extractor. The term $H(Z_\Delta )$ captures the entropy of these features, ensuring they are diverse and contain sufficient information for the target task.

TransRate evaluates two main aspects of feature representation:

- Completeness: A high value of $H(Z_\Delta )$ indicates that the features provide a rich representation of the input space, which correlates with better performance on the target task. This aspect measures how well the features represent different classes.
- Compactness: The term $H(Z_\Delta | Y)$ assesses the compactness of the features within each class. A lower value suggests that features from different classes are well-separated, thereby improving generalization performance. This ensures that features within each class exhibit similarity.

The final TransRate score balances these two aspects, intending to select models that achieve the highest values of $TrRTs \rightarrow Tt(g)$. This selection process is essential for determining which PTMs are most suitable for a given task.

### Gaussian Bhattacharyya coefficient (GBC)

The Gaussian Bhattacharyya Coefficient (GBC)^13^ maps images from the target dataset into a space defined by the features extracted from the source model. In this space, each class in the target dataset is represented by a Gaussian distribution. GBC quantifies how effectively the classes can be distinguished from each other using the Bhattacharyya coefficient. A small overlap between Gaussian distributions suggests that the knowledge from the source model is useful for the target task and should transfer effectively, while a large overlap indicates difficulty in separating target classes, implying poor transferability.

The rationale for using the GBC is that the clear separability of target classes in the source feature space enhances the effectiveness of the source model on the target task. Empirical evaluations demonstrate that GBC provides a robust method for predicting transferability, showing significant correlations between the calculated scores and the actual accuracies achieved after finetuning the models on various target datasets. This is evidenced by superior rank correlations compared to existing transferability metrics.

For practical implementation, covariance matrices are often approximated as diagonal or spherical due to limited sample sizes per class, enhancing numerical stability and computational efficiency. Additionally, GBC offers a theoretical upper bound on the optimal Bayes classification error when only the classification head is finetuned. However, this guarantee becomes less certain in full network finetuning scenarios. Nevertheless, empirical results confirm that GBC remains a valuable predictor of transferability, effectively guiding the selection of the best source model for target tasks.

### Label-feature correlation (LFC)

LFC^14^ evaluates the similarity of features within the same class in the target dataset. It provides an analytical framework for transferability estimation by linearizing the model around its pretraining point. It introduces two baselines for model selection: Label Gradient and Label-Feature Correlation. LFC estimates transferability by calculating the correlation between the target task labels and the features (or activations) of the pretrained model. This correlation is obtained by running the target task through the pretrained model to get the feature representations for each input and then computing the correlation between the target task labels and these representations. The intuition is that if the target task labels are strongly correlated with the features, then finetuning this model to the target task should perform well, as the features already capture relevant information for the target task. LFC shows a moderate improvement in large-size data scenarios, but it performs well in scenarios with limited data (or the few-shot transfer).

### Probably approximately correct transfer (PACTran)

PACTran^15^ criticizes LEEP and $\mathscr {N}$LEEP for focusing too much on how well source models generalize and not enough on their performance with new target tasks. Instead of directly assessing the training error on the source dataset, PACTran employs a linear model with a “flatness regularizer” to enhance generalization. This model is optimized to map features to target labels using a specific optimization technique.

The PACTran framework offers two key advantages: First, it is grounded in learning theory, particularly using PAC-Bayesian bounds to quantify the generalization gap. This distinguishes it from LEEP, which relies on information theory. Second, it is suitable for classification tasks due to its usage of cross-entropy loss, unlike the H-score with the least squares solution.

**Fig. 2:**
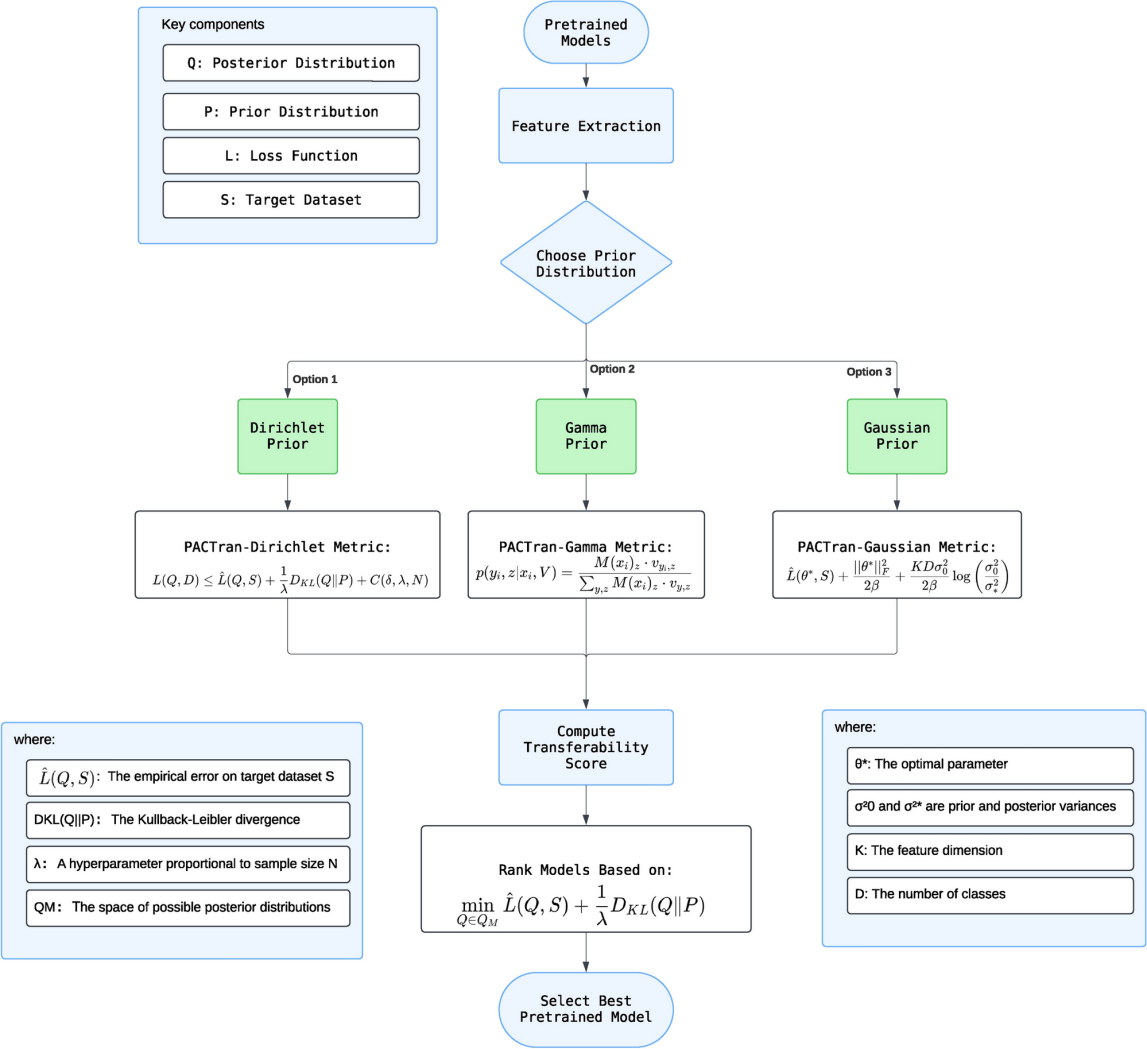
Calculating the PACTran score.

Moreover, PACTran offers three distinct distribution frameworks: PACTran-Dirichlet, PACTran-Gamma, and PACTran-Gaussian. These frameworks provide flexibility in modeling distributions and allow for more specific approaches for different tasks. Figure 2 demonstrates the flowchart illustrating the key components and steps involved in the PACTran framework.

### Self-challenging fisher discriminant analysis (SFDA)

The rationale behind SFDA (Self-challenging Fisher Discriminant Analysis) is to create a robust framework for ranking and selecting PTMs for transfer learning. SFDA focuses on maximizing class separability and enhancing model performance through a self-challenging process that helps identify the best-performing model for a specific target task. SFDA^16^ differs from previous methods like LEEP, $\mathscr {N}$LEEP, and LogME by focusing on the dynamics of the finetuning process rather than just static representations.

SFDA is built on the Regularized Fisher Discriminant Analysis (Reg-FDA) concept. Initially, SFDA projects static features into a Fisher space to enhance class separability, akin to the effect of finetuning. This transformation helps maximize the separation between classes while minimizing intra-class variability.

1. Feature projection (Reg-FDA): To enhance class separability, SFDA uses a projection matrix *U* to transform the input features: $\tilde{x} = U^T \hat{x}$, where $\hat{x} \in \mathbb {R}^D$ are the original features, and $\tilde{x} \in \mathbb {R}^{D'}$ are the transformed features in the Fisher space. The projection matrix *U* is optimized to: maximize $|U^T S_B U|$ and minimize $|U^T S_W U|$, where $S_B$ and $S_W$ are the between-class and within-class scatter matrices, respectively. Regularization is applied to $S_W$ to avoid overfitting, ensuring that the transformed features provide better class separability.
2. Self-challenging mechanism: SFDA then employs a self-challenging mechanism to increase the classification difficulty and push models to better handle challenging cases. This mechanism modifies input features using noise augmentation: $\bar{x}_n = p_n \hat{x}_n + \left( 1 - p_n\right) \mu _c$, where $p_n$ is the confidence score for the *n*-th sample’s prediction, reflecting how confidently the model classifies the sample, and $\mu _c$ represents the mean of feature vectors from classes other than the true class $y_n$, introducing contrast and making classification harder. By increasing the difficulty of classification, this mechanism encourages PTMs to improve their robustness and performance on harder examples, better capturing the dynamics of finetuning.
3. Transferability score: The final step involves calculating a transferability score $T_m$ for each pretrained model *m* to assess its performance on the target task:
  6 $$\begin{aligned} T_m = \sum _{i=1}^N \log p\left( y_i | x_i; \theta _m, h_m\right) \end{aligned}$$
  where $p\left( y_i | x_i; \theta _m, h_m\right)$ is the probability of the model correctly predicting label $y_i$ given input $x_i$, and $\theta _m$ and $h_m$ represent the feature extractor and classification head of the model, respectively.

This transferability score helps rank the PTMs by considering their ability to separate classes effectively and handle difficult examples. This comprehensive approach ensures that the chosen model performs well not only on easier cases but also in more challenging ones, facilitating robust transfer learning. SFDA evaluates and ranks models by projecting input features, applying noise augmentation for robustness, and computing the transferability score $T_m$. This approach ensures that models are assessed based on their ability to separate classes and their robustness to challenging examples.

### Neural collapse transferability index (NCTI)

The NCTI measures how effectively a pretrained model’s features can be adapted to a target task during finetuning. The rationale for NCTI lies in quantifying specific aspects of feature representations to assess transferability across PTMs, as follows:

- How tight the groups are (within-class variability): A good model clusters similar items closely together, resembling the organization of similar objects into boxes. Tighter groupings of features within each class correspond to higher scores. The within-class covariance is calculated as:
  $$\Sigma _c = \frac{1}{n_c} \left( H_c - \mu _c\right) ^\top \left( H_c - \mu _c\right) ,$$
  where $H_c$ represents the features for class $c$ and $\mu _c$ is their average. The within-class variability score is defined as:
  $$S_{\text {vc}}(H_m) = -\sum _{c=1}^{C} ||Z_{mc}||_*,$$
  with $Z_{mc}$ being the logits from model $m$ for class $c$.
- How smooth the transitions are (simplex encoded labels): Effective models maintain smooth transitions between categories, akin to a color gradient. This smoothness aids in understanding relationships among classes. The SEL matrix is defined as:
  $$\hat{Z}[c,i] = {\left\{ \begin{array}{ll} 1 - \frac{1}{C} & \text {if } c = y_i \\ -\frac{1}{C} & \text {if } c \ne y_i \end{array}\right. }$$
  where $y_i$ is the actual label for sample $i$. The SELI score is given by:
  $$s_{\text {eli}}(H_m) = ||H_m||_*,$$
  indicating how well the features fit this structured transition.
- How good at classification (nearest centroid): This aspect relates to having a reference example for each category, allowing the model to compare new items to these reference points. Improved matching of centroids results in more accurate classifications. The probability of a feature belonging to class $c$ is expressed as:
  $$\log P(y=c|h) = -\frac{1}{2} \left( h - \mu _c\right) ^\top \Sigma ^{-1} (h - \mu _c) + \log P(y=c),$$
  where $h$ is a feature vector and $\mu _c$ is the class centroid. The normalized probability is calculated as:
  $$z_{mi,c} = \frac{\exp \left( \log P\left( y=c|h_{mi}\right) \right) }{\sum _{k=1}^{C} \exp \left( \log P\left( y=k|h_{mi}\right) \right) },$$
  leading to the final nearest centroid score:
  $$S_{\text {ncc}}(H_m) = \frac{1}{N}\sum _{i=1}^{N} z_{mi,y_i}.$$
  The Final NCTI score: The final NCTI score aggregates these components, normalized to the [0,1] range:

7 $$\begin{aligned} S_{\text {total}} = S_{\text {vc}}(H_m) + s_{\text {eli}}(H_m) + S_{\text {ncc}}(H_m). \end{aligned}$$

Higher scores (closer to 1) indicate that the model is likely to adapt better to new tasks, require less finetuning, and be a more suitable choice for transfer learning. In the selection process for different PTMs, calculating their NCTI scores and choosing the one with the highest score will likely lead to better performance when adapting to a new task.

### Efficient multi-task model selector (EMMS)

In traditional methods, labels are typically represented using either one-hot coding or embedding layers. Instead, EMMS^18^ adapts a textual approach, which makes it adaptable to various tasks beyond just image classification.

Traditional methods for representing labels, such as one-hot encoding, have limitations in capturing the rich semantic relationships present in label data. To address this, EMMS leverages foundation models, such as Contrastive Language-Image Pretraining (CLIP), Bidirectional Encoder Representations from Transformers (BERT), and Generative Pretrained Transformer 2 (GPT-2), to transform various label formats (text captions, bounding boxes) into unified vector embeddings. This transformation enhances the model’s ability to generalize across tasks by utilizing the semantic context encoded within these embeddings, providing a more robust basis for multi-task learning.

- Label embedding with foundation models: By utilizing the semantic relationships learned by foundation models, EMMS effectively implements label embeddings that capture rich semantic connections encoded in text. For example, CLIP can learn meaningful relationships between images and text, enabling EMMS to predict labels for tasks without specific training, thus extending its applicability across various domains.
- Probabilistic framework: Furthermore, EMMS incorporates a probabilistic framework that allows for the modeling of uncertainty and noise in label embeddings and features. Real-world data often contains noise, which can adversely affect model performance. By acknowledging this noise in the feature-label mapping, EMMS enhances its robustness and reliability, ensuring effective performance despite variations in data quality.
- Log-Likelihood computation: The log-likelihood function provides a formal mathematical basis for evaluating how well the model fits the observed data. By maximizing the log-likelihood, EMMS quantitatively assesses the performance of different PTMs, helping to rank models based on their expected performance and enabling a more informed selection process.
- Weighted linear square regression (WLSR): WLSR is employed to estimate the transferability of PTMs by establishing relationships between model features and label embeddings. This approach simplifies the optimization problem while accounting for the contributions of various model features to the final output. The flexibility of WLSR allows EMMS to accommodate the nuances of different tasks while maintaining computational efficiency.
- Fast alternating minimization algorithm: Finally, the fast alternating minimization algorithm is crucial for efficiently optimizing the model parameters. By iteratively updating the weights and label embeddings, this algorithm ensures that the optimization converges to a solution that minimizes the transferability score. The method is efficient and guarantees convergence, making it suitable for scenarios with large datasets and complex models.

**Fig. 3:**
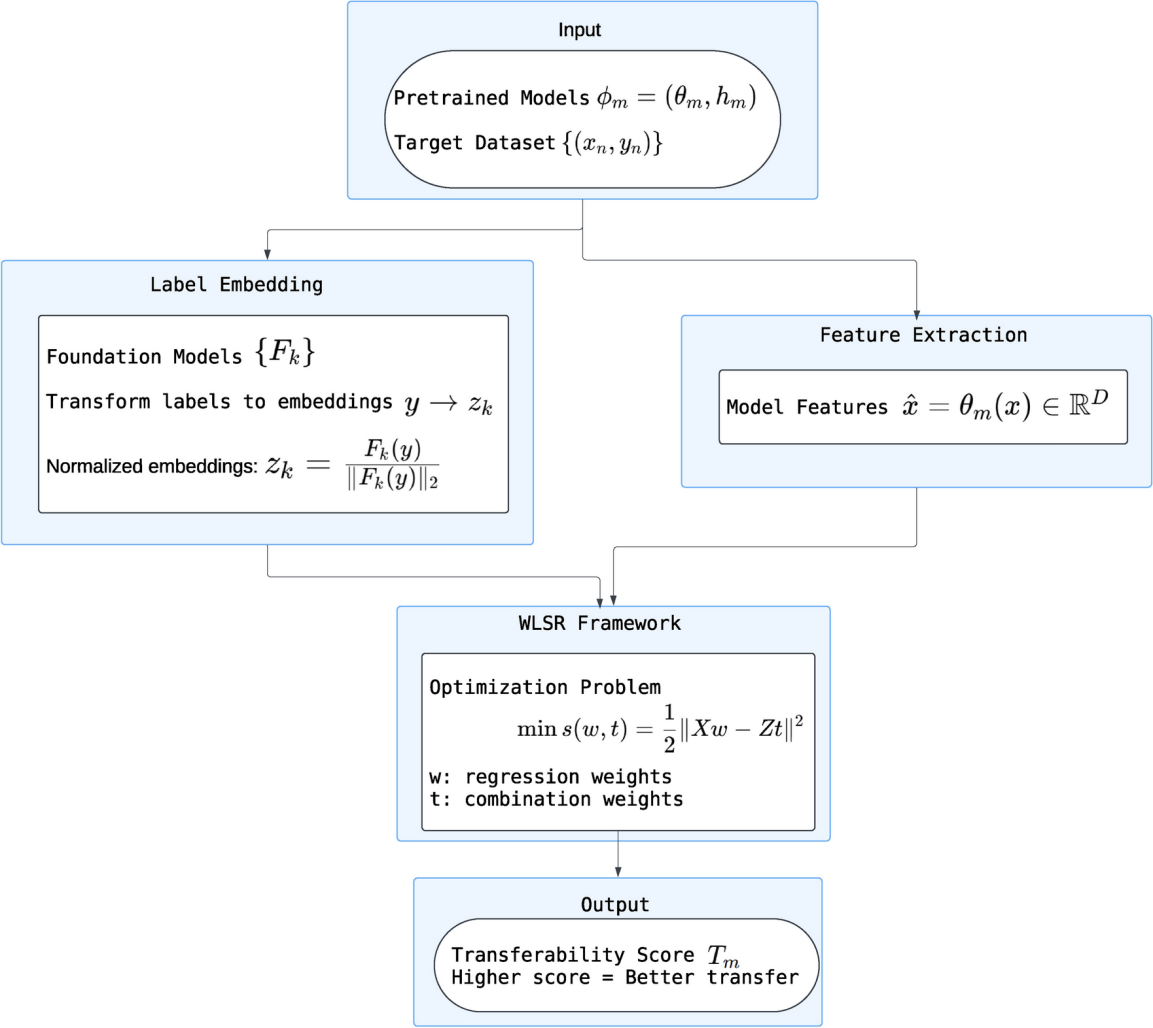
Calculating the EMMS score.

Figure 3 visualizes the key components and steps involved in the EMMS framework, further elucidating the interaction between input features, foundation model embeddings, and the resulting transferability rankings.

Thus, EMMS serves as a multitask model selector by providing a more efficient transferability metric, addressing challenges of varying model performance across different tasks, and minimizing computational resource requirements for adaptation.

### Energy-based transfer (Etran)

Traditional methods for assessing transferability rely on features extracted from PTMs, which can vary significantly before and after finetuning. This variability often leads to unreliable and domain-independent assessments, complicating effective model selection. To overcome these limitations, Etran introduces a novel metric that distinguishes whether a target dataset is IND or OOD^19^.

Etran aims to streamline the selection of optimal PTMs for transfer learning tasks such as object detection and image classification. By focusing on the IND/OOD distinction, Etran enhances the accuracy of performance predictions after finetuning, thus addressing the discrepancies in source and target dataset distributions that traditional methods struggle with.

This metric is efficient and applicable across classification, regression, and object detection tasks, marking a significant advancement in the literature. It integrates classification and regression scores through techniques like Linear Discriminant Analysis (LDA) and Singular Value Decomposition (SVD), demonstrating empirical success by outperforming existing methods in object detection and classification.

The overall transferability score $T$ is computed as

$$T = S_{en} + S_{cls} + S_{reg},$$

where $S_{en}$ represents the energy score, estimating the likelihood of the target dataset being IND or OOD, derived from energy-based models (EBM). The classification score $S_{cls}$ evaluates the model’s accuracy in classifying data after finetuning, while the regression score $S_{reg}$ is primarily applicable to object detection, assessing the model’s ability to accurately predict bounding boxes.

The energy score $S_{en}$ is calculated based on the energy function $E(x) : \mathbb {R}^D \rightarrow \mathbb {R}$, with the probability density $p(y|x)$ expressed via the Gibbs distribution:

$$e^{-E(x,y)} = \frac{p(y|x)}{R_{y'} \sum e^{-E(x,y')}}$$

where the partition function $R_{y'} = \sum e^{-E(x,y')}$ normalizes these probabilities, leading to the Helmholtz free energy $E(x)$:

$$E(x) = -\log R_{y'}$$

After computing $S_{en}, S_{cls},$ and $S_{reg}$, the overall score $T$ allows for ranking PTMs, with higher scores indicating better-anticipated performance post-finetuning.

In addition to their primary role in model selection for classification tasks, certain transferability metrics offer supplementary functionalities. For example, LogME has a general capability for supervised and unsupervised PTMs across classification, regression tasks, and multi-modality for vision and language. Similarly, TransRate allows the selection of the best-pretrained layers to enhance transfer learning outcomes. GBC extends its utility beyond model selection to assist in choosing target datasets for classification and segmentation tasks. SFDA can select multiple PTMs for the best model ensemble. Additionally, EMMS provides a generic multi-modality selection task, covering image classification, annotation, comprehension, and landmark detection. Lastly, ETran offers comprehensive model selection capabilities across classification, regression, and object detection tasks.

## Categorization of transferability scores

**Fig. 4:**
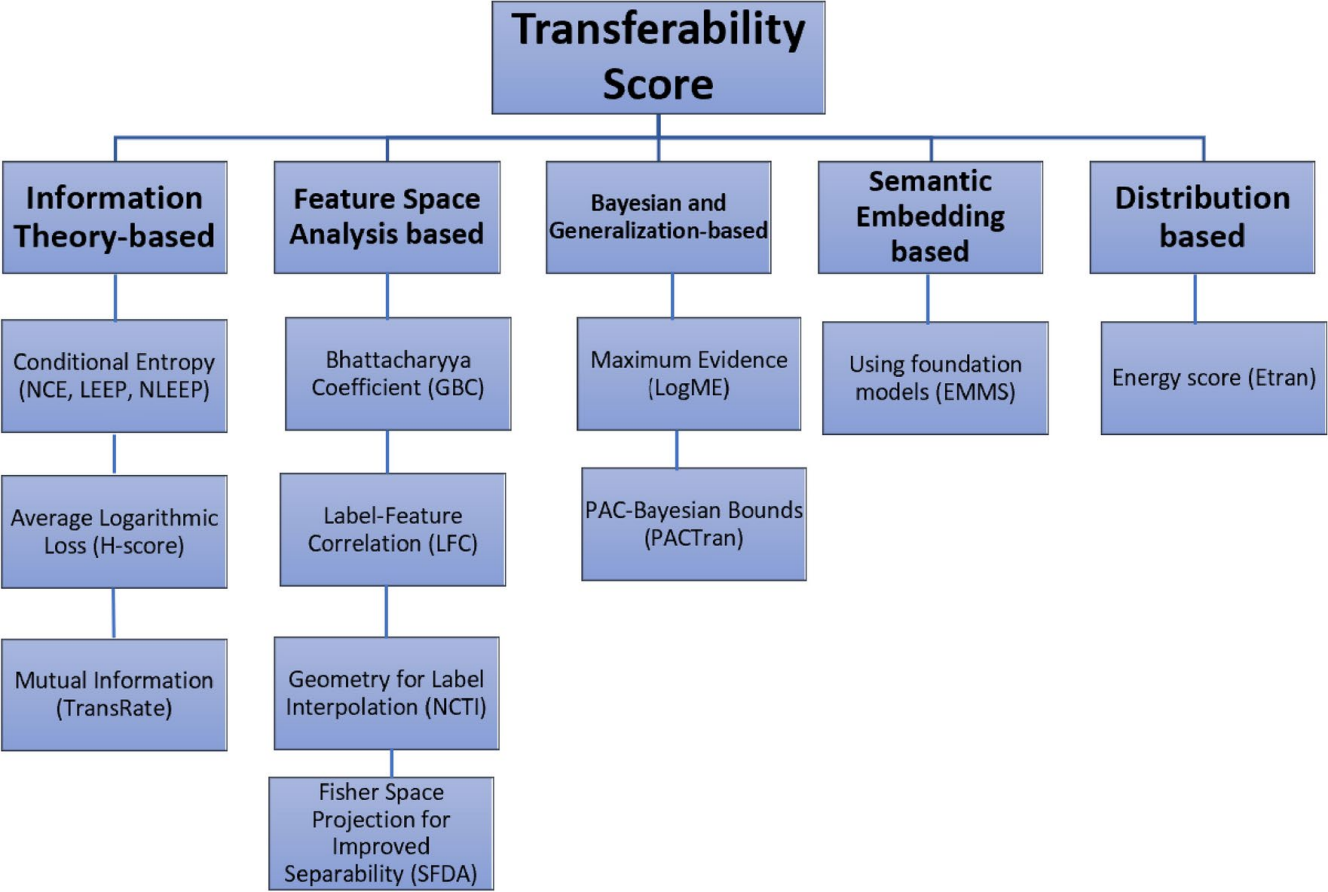
The categories of transferability scores.

After reviewing various transferability scores, they can be grouped into the following categories, as shown in Fig. 4. While these categories help to organize the different approaches to transferability assessment, some metrics are based on multiple theoretical foundations and could fit into multiple categories. This categorization focuses on the primary theoretical basis of each metric while recognizing its complex nature.

1. Information theory-based scores: They primarily use concepts from information theory to evaluate transferability by analyzing the relationship between pretrained features and target labels. These scores include methods such as conditional entropy (NCE, LEEP, and $\mathscr {N}$LEEP) and average logarithmic loss (H-score). While their main focus is on information theory, the H-score also considers feature space analysis through covariance matrices and class means, which can be related to feature space analysis. In addition, TransRate primarily falls into this category due to its use of mutual information and coding rate based on information theory. Still, it also evaluates feature completeness and compactness, which can also be related to the feature space analysis.
2. Feature space analysis-based scores: These scores focus on analyzing the feature representations learned by the pretrained model. They evaluate how well these features can separate or discriminate the classes in the target dataset. The methods include using the Bhattacharyya coefficient (GBC), calculating the label-feature correlation (LFC), assessing within-class variability and structuring geometry for label interpolation (NCTI), and projecting static features into a Fisher space to improve separability (SFDA).
3. Bayesian and generalization-based scores: These scores employ Bayesian techniques or learning theory principles to estimate how well the pretrained model will generalize to the target dataset, such as maximum evidence (LogME), and PAC-Bayesian bounds (PACTran).
4. Semantic embedding-based score: The EMMS score employs label embeddings from the foundation models to capture semantic relationships between labels to estimate transferability.
5. Distribution-based score: The ETran score examines whether the target dataset is IND or OOD compared to the training data of PTMs.

## Methodology

This study presents a systematic framework for evaluating transferability scores in selecting optimal PTMs for transfer learning in image classification tasks. The methodology builds on established approaches in the transfer learning literature while providing a comprehensive evaluation framework that spans CNN and ViT architectures.

### Dataset selection and characteristics

The experimental framework uses a set of benchmark datasets that have been consistently used in previous transferability studies. These datasets were selected to maintain consistency with previous research while ensuring comprehensive coverage of different visual recognition challenges.

The datasets are grouped into fine-grained classifications (FGFC Aircrafts^35^, Stanford Cars^36^, Food101^37^, Oxford IIIT Pets^38^, Oxford-102 Flowers^39^), a coarse-grained classification (Caltech101^40^, Cifar10^41^, Cifar100^41^, and VOC2007^42^), a texture classification (DTD)^43^, and a scene classification (SUN397)^44^.

The datasets represent a significant diversity in both scale (ranging from 8,189 images in Oxford-102 Flowers to 101,000 images in Food-101) and complexity (ranging from 10 classes in Cifar10 to 397 classes in SUN397), allowing for a robust evaluation of transferability metrics across different problem domains. More details on the datasets can be found in the Online Appendix Table S1.

### Model selection and architectures types

This study includes PTMs from the CNN and ViT families, following the established standard in model transferability research. This study tested various model CNN model architectures and depths, including Inception (Inception$\_$v3, GoogleNet), ResNets (ResNet34, ResNet50, ResNet101, ResNet152), DenseNets (DenseNet121, DenseNet169, DenseNet201) and MobileNets family (MobileNet$\_$v2, MnasNet1_0), and 10 ViT models (DeiT$\_$base, DeiT$\_$tiny, DeiT$\_$small, DINO$\_$small, Mocov$3$ $\_$small, Pvtv$2$ $\_$b2, Pvt$\_$tiny, Pvt$\_$small, Pvt$\_$medium, Swin$\_$t) that are pretrained on the ImageNet dataset. The study benchmarks these metrics on image classification only.

This selection of models, consistent with previous transferability studies, ensures fair comparisons with existing literature while providing insight into model-specific transfer learning characteristics.

### Ground truth estimation

To determine the ground truth for test error for each pretrained model on each target dataset, all PTMs were finetuned on each target dataset using a brute-force approach. This approach identifies the best achievable accuracy for each combination of the target dataset and model. While the practical use of transferability scores does not require this step, it remains crucial for the evaluation of these scores, as demonstrated in this work. For CNN PTMs, two sets of ground truths are considered: GT1, based on the ground truths introduced in the ImageNet weights correlation paper^34^, and GT2, re-calculated using the EMMS score^18^. The average absolute percentage change between the two ground truths is $7.78\%$, with further details provided in Online Appendix Tables S2, S3, and Figure S1. Additionally, the ground truths for the ViT pretrained model are also calculated according to the EMMS paper^18^.

### Evaluation protocol

The evaluation protocol maintains a separation between training and test data, with test splits reserved exclusively for the validation of transferability scores. This ensures an unbiased assessment of the transferability metrics across all PTMs in the tested pool. To evaluate the effectiveness of these metrics, the weighted Kendall-Tau correlation coefficient is used to compare the alignment of the rankings derived from each transferability score with those from the ground truth experiments. This statistical measure provides a quantitative assessment of the ability of each score to correctly rank PTMs from best to worst performing, a process that will be further detailed in the next section.

### The proposed framework

The framework selects the optimal pretrained model for image classification tasks based on transferability scores. It evaluates the performance of different PTMs and provides a systematic approach to model selection. The process is organized as follows:

1. Input: The methodology begins by collecting a pool of PTMs, which includes CNN and ViT architectures. These models, initially trained on ImageNet, provide the basis for the transfer learning experiments.
2. Feature extraction: For each target dataset, feature extraction is performed on all PTMs in the pool. The target dataset is passed through the architecture of each model, capturing activation patterns from the penultimate layer. These features are then stored for later analysis.
3. Transferability score evaluation: Calculate transferability scores using the extracted features and target dataset labels. Scores applied include NCE, H-score, LEEP, $\mathscr {N}$LEEP, LogME, TransRate, GBC, LFC, PACTran, SFDA, NCTI, EMMS, Etran, and ImageNet weights.
4. Ranking of models: Rank the PTMs based on their transferability scores using the weighted Kendall-Tau correlation coefficient, with higher scores indicating a better potential for transfer learning performance.
5. Computational efficiency analysis: Evaluate the computational cost associated with each transferability score to determine their efficiency.
6. Optimal model selection: The final selection process integrates multiple factors including the transferability score rankings, the computational efficiency metrics, and the specific requirements of the target task.
7. Output: The final selected model is presented along with its expected performance.

**Fig. 5:**
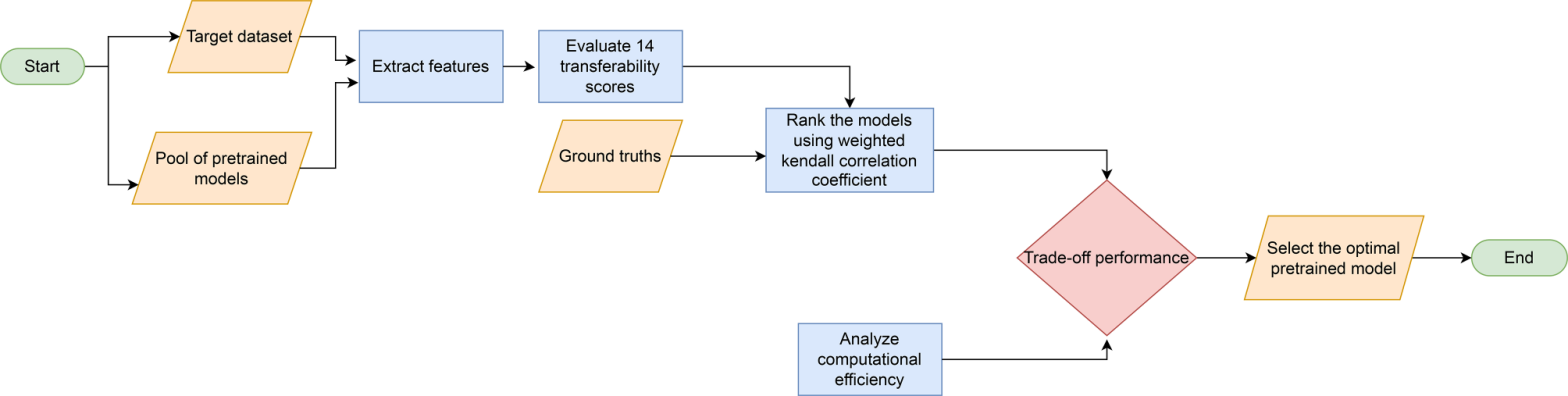
The flowchart of the proposed framework.

Figure 5 shows the flowchart of the proposed framework and its operational processes. To further clarify the proposed framework, a pseudo-code representation of the model selection process can be found in the Online Appendix algorithm S1.

## Metrics for assessing transferability scores

After the introduction of transferability scores, multiple metrics are proposed to evaluate their effectiveness. This involves comparing the ground truths of the tested datasets with the corresponding PTMs to assess the performance of these scores. Several assessing metrics are introduced in the papers, including:

1. Pearson correlation coefficient: This is a classic metric for assessing the correlation between two vectors. It evaluates how well the transferability scores correlate with the ground truths. However, it is sensitive to outliers, and a low correlation does not necessarily indicate poor performance, as it may not accurately capture nonlinear relationships. However, the correlation value should be correct despite the nonlinear relationship^45^.
2. Kendall-Tau correlation coefficient: This metric focuses on measuring the ordinal association, capturing the strength and direction between a transferability score and the ground truths. It prioritizes the order of the scores over their exact values, which is crucial for evaluation purposes. Kendall-Tau is robust to outliers and does not assume linearity between the tested vectors. However, it treats all PTMs equally, which may not address differences in model performance effectively^46^.
3. Weighted Kendall-Tau correlation coefficient: Building on the Kendall-Tau Correlation Coefficient, this metric introduces weighting to emphasize top-performing PTMs. It aims to prioritize the best-performing PTMs by assigning them higher weights^47^. This is particularly useful when the primary goal of transferability estimation is to rank PTMs and select the best one.
4. Relative Top-1 Accuracy: It measures the closeness of the highest transferability score to the ground truths. It involves ranking the transferability metrics in descending order and dividing the highest transferability score by the best finetuning accuracy of the corresponding pretrained model. Some metrics also apply this approach to the relative top-3, ranking the top three transferability metrics instead of just one^10^.

The weighted Kendall-Tau correlation coefficient is chosen as the primary metric for evaluating transferability scores because it has several advantages. First, unlike the Pearson correlation coefficient, which can be biased by outliers and doesn’t capture nonlinear relationships well, the Weighted Kendall-Tau is more robust in these situations. Second, it introduces a weighting mechanism that prioritizes the best-performing models, which is critical for assessing transferability. By giving more weight to the most promising models, it ensures that the rankings reflect the models most likely to be selected in practice. Third, Weighted Kendall-Tau maintains stability while accurately ranking models, even in the presence of outliers. This makes it a more reliable and comprehensive metric than others, such as Relative Top-1 Accuracy, which focuses only on the best performer and misses the broader relationships between models. The weighted Kendall-Tau captures these distinctions, making it the superior choice for evaluating transferability scores.

## Evaluation of transferability scores

The transferability scores obtained in this study may differ from those reported in the original papers due to variations in experimental setups, which can significantly affect the results. To ensure consistency in the experimental environment, this study re-calculated all transferability scores. Differences arose from several factors: Firstly, variations in the choice of datasets, as not all metrics in the original papers tested the same datasets used as benchmarks here. Secondly, differences in the model pool selection, where some models were not widely tested across the same PTMs or were limited in number. Thirdly, variations in score assessment methods contributed to the observed differences, with some studies using only the Pearson correlation coefficient while others employed additional metrics.

It is important to mention that the scores for $\mathscr {N}$LEEP and PACTran are non-deterministic and vary significantly for every execution. The values presented are averages derived from four execution runs. This variability arises due to the nature of these methods. $\mathscr {N}$LEEP uses checkpoints and behaves similarly to feature extraction from the penultimate layer, while the optimization process of PACTran starts from random parameter initialization, resulting in diverse outcomes. PACTran’s optimization aims for local minima and depends on the initial starting point, which can vary due to its iterative nature and the inclusion of a flatness regularizer. Consequently, the final results depend on the initial starting point, which is not fixed.

### Analyzing correlation in transferability estimation

**Fig. 6:**
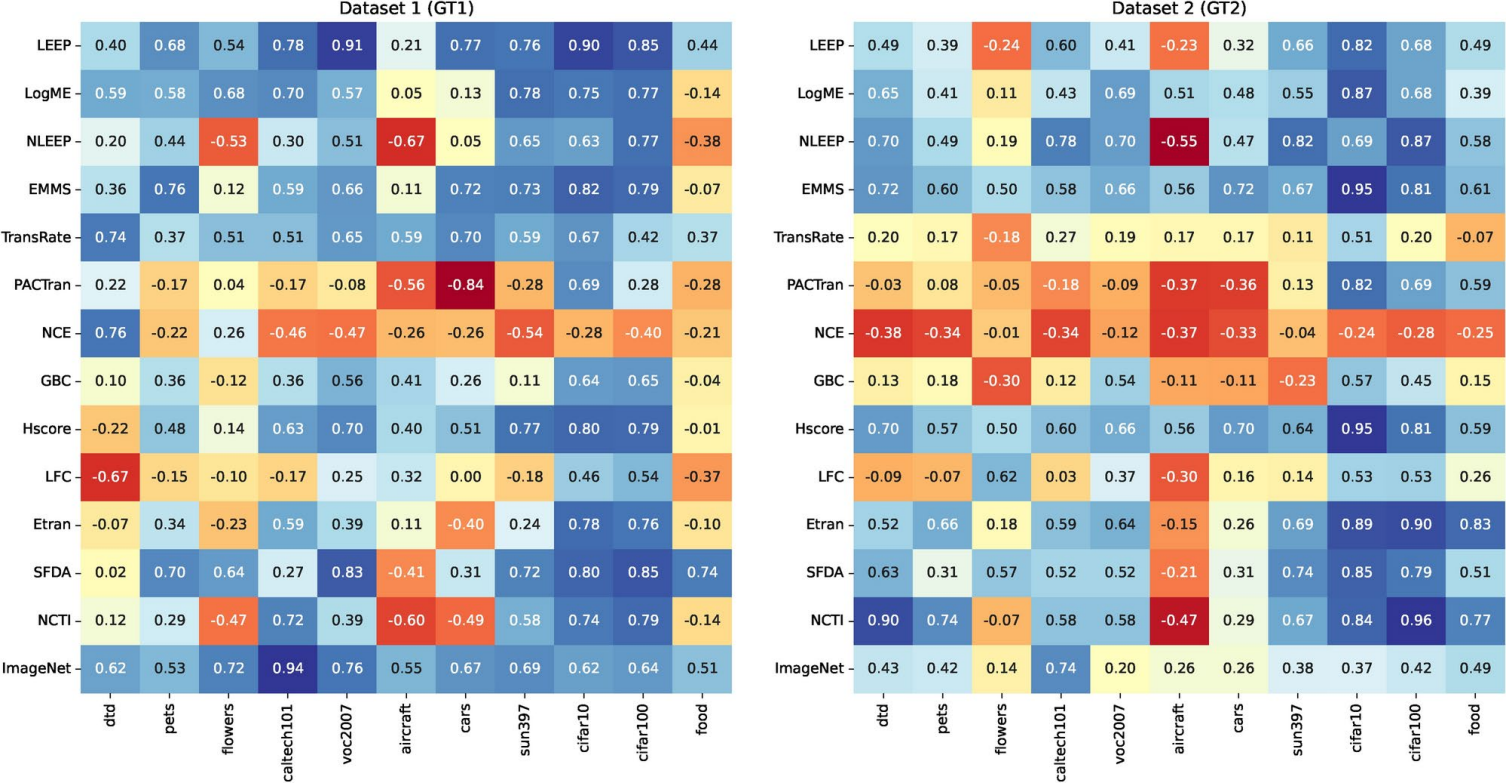
The heatmaps of the weighted Kendall-Tau correlation coefficients for the transferability scores of all tested datasets using two ground truths for CNN PTMs.

Figure 6 illustrates heatmaps displaying the weighted Kendall-Tau correlation coefficients for the transferability scores across all tested datasets. The heatmaps illustrate that most transferability scores have varying degrees of performance in GT1 and GT2. Figure S2 in the Online Appendix shows the radar chart for the top-performing scores only for each dataset for CNN PTMs for both ground truths. Despite this instability of behavior and sensitivity to the values of the ground truths, this study tries to correlate some conclusions on the tested metrics. A positive correlation is considered acceptable if it is above 0.5. Correlations exceeding 0.5 for weighted Kendall-Tau are categorized as good, while those surpassing 0.75 are considered very good. The $x-$axis displays datasets arranged from the smallest to the largest number of images, while the $y-$axis shows the transferability scores arranged chronologically based on their appearance in the research timeline. To avoid the sensitivity of the ground truths, the following discussion focuses only on the mutual behavior of GT1 and GT2.

- *The performance of information theory-based scores* The performance of LEEP and $\mathscr {N}$LEEP is influenced by the number of images in the dataset. Better scores are typically observed with a larger number of images, except for the Food dataset. This happens because the empirical conditional distribution is prone to overfitting, particularly when there are fewer images per class. Despite the large size of the Food dataset, LEEP’s reliance on pseudo-labels generated by a pretrained model can introduce potential inaccuracies. The pretrained model may not have sufficiently learned the detailed features needed to accurately distinguish between different food dishes, leading to noisy or inaccurate pseudo-labels. H-score demonstrates consistently good to very good performance across most datasets, except for fine-grained datasets such as Flowers, Aircrafts, and Cars, where its performance is acceptable. This difference arises from the complicated nature of these datasets, characterized by visually complex images with complicated patterns, textures, and shape/color variations. The H-score depends on covariance matrices and class means to quantify the separation between classes in the feature space. However, in datasets with a small number of representations relative to the number of features, these measures may not accurately capture the differences between fine-grained classes. Furthermore, reliance on class-conditional means and covariance matrices makes the H-score prone to overfitting, as it may capture noise rather than true predictive patterns, leading to difficulties in effectively separating the classes. For NCE, there is a negative correlation across all datasets except for DTD. Interestingly, it demonstrates the highest transferability score for the DTD dataset compared to other metrics. The DTD dataset involves texture classification, a different domain from typical image classification tasks, and presents a challenge for direct transferability from ImageNet features. NCE assumes similarity between the source and target tasks in input data and differs only in label assignments. This assumption is more appropriate with the nature of the DTD dataset, where textures can be considered as low-level visual attributes across various object labels. Moreover, unlike other transferability metrics, NCE does not rely on the relationship between source and target task classes. This may be advantageous for DTD, where texture classes are unlikely to correspond directly to ImageNet object categories. Lastly, TransRate proves sensitive to the stability of ground truths labels and struggles to bring consistent behavior and draw definitive conclusions. Even minor alterations in ground truth labels can substantially impact sample class assignments, consequently altering class conditional coding rates. This instability makes TransRate less reliable as a metric for transferability estimation, particularly when ground truths are subject to minor modifications.
- *The performance of the feature space analysis-based scores* GBC shows strong performance on datasets with a small number of classes, such as Cifar10 and VOC2007. However, it struggles on datasets with high-dimensional matrices, like Cifar100, due to the instability of Bhattacharyya distance calculations^48^. Similarly, LFC performs well on Cifar10 but struggles on complex datasets with overlapping classes or less distinct features, where linear correlations between features and labels are weaker. NCTI generally performs acceptably across most datasets but underperforms on small, fine-grained datasets like Airplanes, Cars, Pets, and Flowers. The PCA step may fail to capture relevant discriminative features in these datasets, leading to difficulties in effectively training LDA to separate visually similar classes. SFDA demonstrates strong performance across datasets, especially excelling with larger datasets. Its ConfMix operation, which mixes features with class conditional probabilities and outer class means, proves highly effective in capturing the dynamics of finetuning, particularly on challenging examples. However, SFDA shows relatively weaker performance on smaller, fine-grained datasets like Cars, Airplanes, Pets, and Caltech. It struggles to fully use its strengths due to the complexities of the class structures or the limited number of examples available. SFDA generally excels because it relies on class-related information and handles tough challenging examples. However, it struggles with datasets with complex class structures or few examples.
- *The performance of the Bayesian and generalization-based scores* LogME performs well to very well, except for Aircrafts, Cars, and Food, which are the smallest and largest fine-grained datasets, because the assumption of LogME of data following a Gaussian distribution may not hold for small fine-grained datasets like Aircrafts and Cars. This also applies to high-dimensional, large fine-grained datasets like the Food dataset. In such cases, where class-conditional distributions are more complex and visually similar, LogME may struggle to capture transferability because of increased overlap in these distributions. On the other hand, PACTran shows a strong correlation primarily with large datasets. It uses PAC-Bayesian bounds to measure the generalization gap, which enables it to perform well on large datasets where there is sufficient data to effectively learn the model parameters.
- *The performance of the semantic embedding-based score* EMMS, generally performs well across datasets but shows weaker performance on DTD, Flowers, Airplanes, and Food. The DTD dataset focused on texture classification, presents a challenge due to the complexity and variability of textures. The foundation models used in EMMS may not have been specifically designed to capture and represent texture features that lead to decreased performance on the DTD dataset. Similarly, fine-grained datasets such as Flowers, Aircrafts, and Food often involve fine-grained classification tasks. The foundation models used in EMMS may not have been trained to capture these fine-grained details, resulting in poor performance on such datasets. Despite the fine-grained characteristics of the Cars and Pets datasets, EMMS performs well. This success is likely because the foundation models used in EMMS (e.g., CLIP, BERT, GPT-2) may have been pretrained on datasets more similar to Cars and Pets, making the transfer and adaptation process more effective.
- *The performance of the distribution-based score* ETran is sensitive to ground truths and struggles to draw definitive conclusions. The small fine-grained datasets with more than 100 classes often feature visually similar categories, and lead to a significant distribution shift from the source dataset. Consequently, ETran faces challenges in accurately determining whether the data is IND or OOD for this dataset type. Additionally, the lack of diversity within these datasets increases the likelihood of examples appearing OOD relative to the training data. Moreover, ETran uses LDA as a classifier, which can be prone to overfitting, especially since LDA assumes that the features extracted by the pretrained model are separable based on the classes of the source dataset. While calculating the energy from the features helps, the distribution shift could still cause difficulties for the energy score component. Ultimately, the sensitivity of Etran to ground truth fluctuations compromises its robustness in determining whether the dataset is IND or OOD.
- *ImageNet accuracy correlation* is highly affected by ground truth values. For GT1, it shows good to very good correlation, whereas the performance on GT2 is poor. Since this score is based only on a direct correlation between the feature representations with labels and the ground truths, it is highly sensitive to variations in ground truth values.

In summary, the type of dataset also plays a role in influencing transferability scores, as summarized in Table 2. While there are exceptions within certain datasets, they have been addressed earlier. In the table, the symbol *X* in the table indicates sensitivity to ground truth variations, which prevents consistent conclusions. To conclude, neither the number of classes alone nor the number of images alone directly determines the performance of the transferability metrics. The table suggests that the performance of different transferability metrics depends on a combination of factors, including the dataset characteristics and domain nature.

**Table 2:** Influence of dataset types on transferability scores.

Transferability score category/ dataset type	Small with large number of classes datasets (Aircrafts, Flowers, Cars, Pets, Sun397)	Large fine-grained datasets (Food)	Texture datasets (DTD)	Simple coarse-grained datasets (Cifar10, Cifar100, VOC2007, Caltech101)
Information theory-based metrics	Struggle due to overfitting on small data	Struggle due to noisy pseudo-labels from PTMs	NCE performs well due to domain shift assumptions fitting texture data	Perform well due to the sufficient data that supports generalization ability
Feature space analysis metrics	Face challenges due to the lack of distinct class separability and high-dimensional feature spaces	X	X	Performs well due to well-separated classes and distinct features
Bayesian metrics	Assume Gaussian distributions that may not hold for complex fine-grained class distributions.	Face difficulties due to complex, overlapping class distributions.	X	Perform well due to better generalization on large data.
Semantic embedding metric	Perform poorly, as pretrained foundation models may not capture fine-grained details well	X	Struggle as PTMs may not be optimized for texture representations	Perform well due to the good alignment and robustness inheritance from foundation models
Distribution-based metric	Struggle due to unreliable energy score estimation and class separability issues on small data	X	X	Perform well due to distribution alignment with ImageNet and the good class separability that supports LDA

**Fig. 7:**
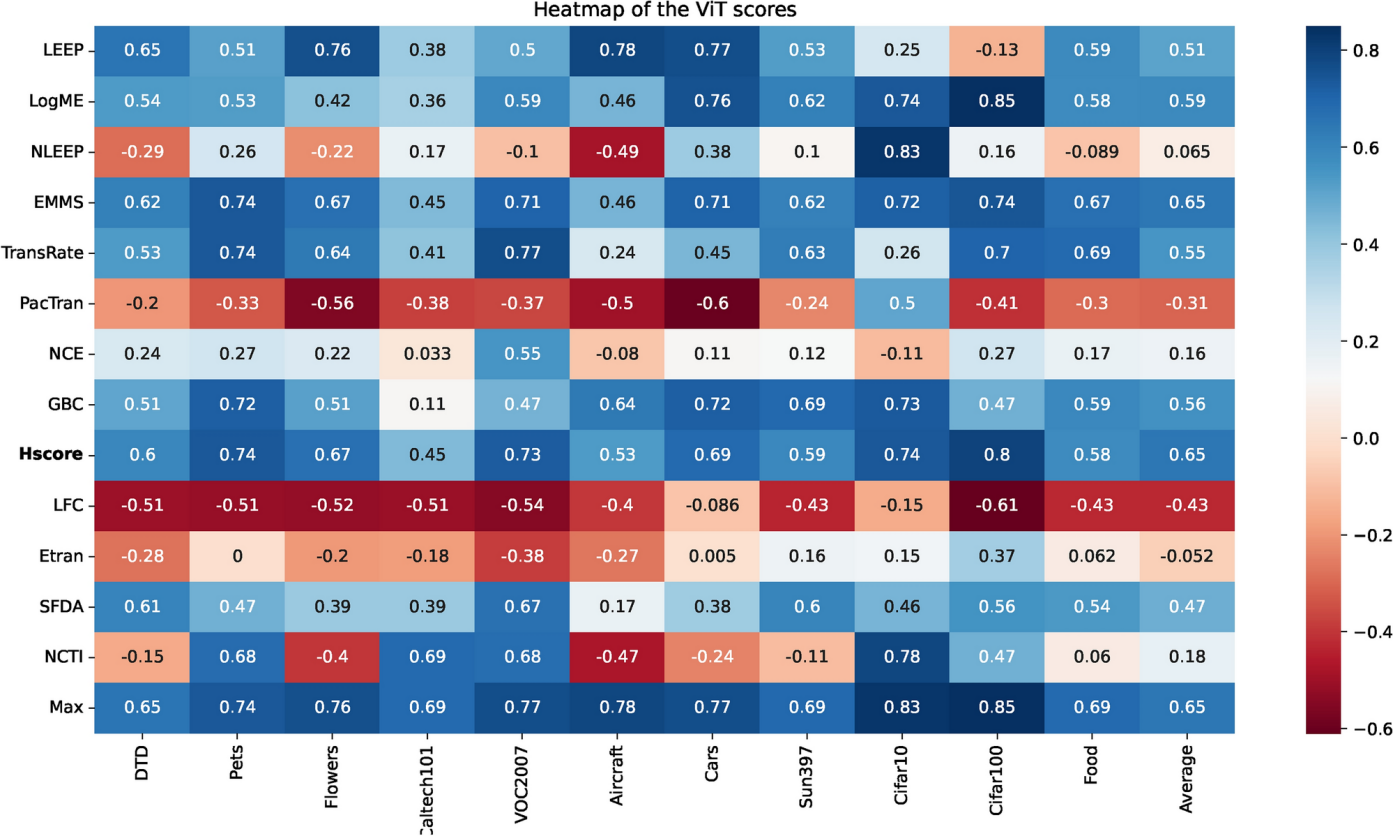
The heatmap of the weighted Kendall-Tau correlation coefficients for the transferability scores of all tested datasets using two ground truths for ViT PTMs.

In addition to evaluating the previously examined CNN pretrained model, this study also assesses the transferability metrics for ViT PTMs. CNN models learn local and spatial features that remain consistent across different image parts through convolutional operations. In contrast, ViT learns global features that consider the entire image context through self-attention mechanisms. Figure 7 shows heatmap results for ViT PTMs, indicating generally better performance of transferability scores with ViTs compared to CNNs. Particularly, transferability scores like LEEP, which relies on pretrained classifiers, benefit from using ViT PTMs on most datasets. Additionally, The performance of GBC differs between CNN and ViT PTMs due to the types of features they learn. CNNs focus on local features, effective for datasets with fewer classes but more complex for those with many classes. ViTs learn global features, which capture extensive context, even for datasets with a large number of classes. However, GBC can effectively estimate the distances from these global representations, enabling it to adapt well to ViT PTMs across datasets of varying class sizes.

ViT is typically trained on larger and more diverse datasets, leading to better generalization and transferability to target datasets. The effectiveness of transferability scores depends on how well source distributions align with target data distributions. When it comes to simple coarse-grained datasets, which involve more distinct object categories that are well-separated, ViT transferability scores also perform well on these tasks, as the classes are relatively easy to distinguish. Yet, similar to CNN transferability scores, the NCE, PACTran, and LFC metrics generally perform poorly even for the ViT PTMs, due to similar reasons.

However, there are significant variations across datasets. For small fine-grained datasets (e.g., Aircraft, Cars, Pets, Flowers, Food), ViTs outperform CNNs due to their ability to capture global, context-aware representations. They effectively extract features used in calculating the transferability scores, due to the self-attention mechanism in ViT. This mechanism helps focus on small but important details that distinguish similar classes. Further details can be found in the Online Appendix Table S4.

Conversely, for the DTD dataset, CNNs with their ability to capture local, spatial patterns, may have an advantage over ViTs. For the DTD dataset, CNNs may have an advantage due to their ability to capture local, spatial patterns. CNNs’ convolutional operations are well-suited for texture classification. ViTs, unless specifically designed or pretrained for texture-related tasks, may struggle with complex textural details. Furthermore, for the scene classification dataset SUN397, CNN PTMs could cover a wide range of scenes and outperform ViTs on this task.

### Analyzing time complexity in transferability estimation

The first step in measuring transferability estimation is to extract source model features for all target datasets for each pretrained model. This process has a time complexity of *O*(*NF*), where *N* is the number of samples and *F* is the feature extraction complexity. Computation time increases significantly in two scenarios: as the number of images grows and when handling high-confidence predictions from the classification head.

**Fig. 8:**
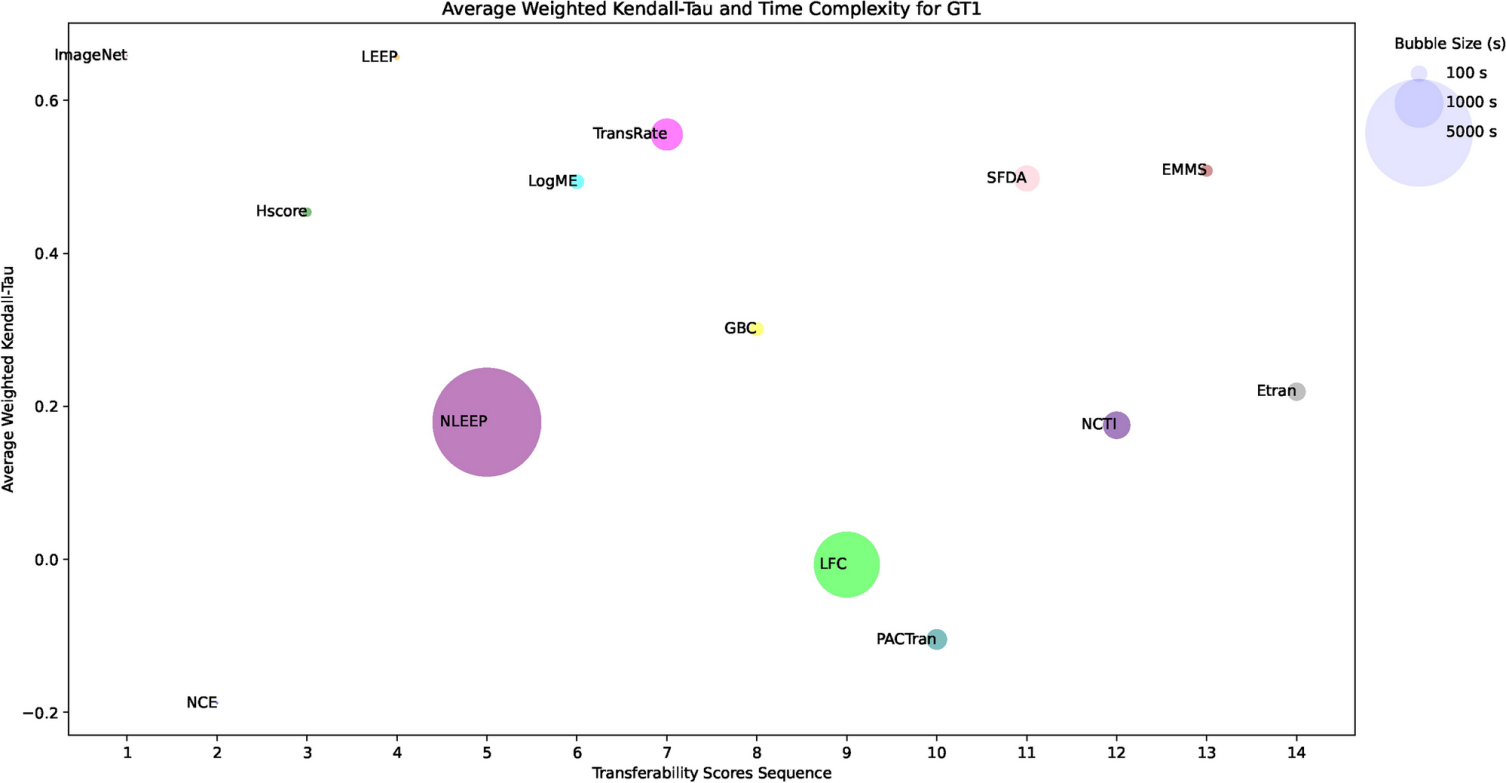
The average weighted Kendall-Tau and the average time complexity among the datasets for all transferability scores for GT1 vs The Timeline Sequence.

**Fig. 9:**
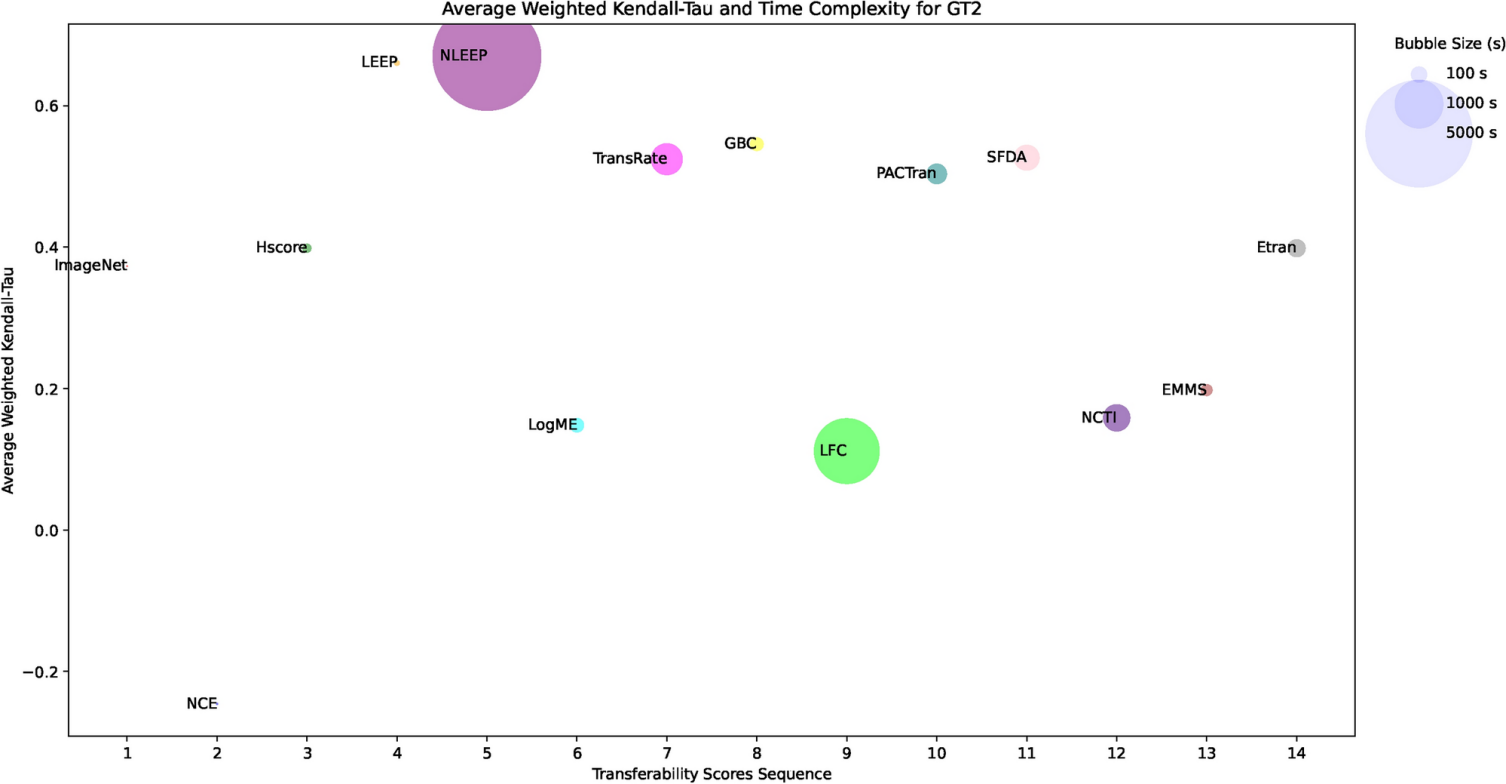
The average weighted Kendall-Tau and the average time complexity among the datasets for all transferability scores for GT2 vs The Timeline Sequence.

The time complexity for all metrics was calculated and averaged per target dataset. Figures 8, and 9 illustrate the average time complexity for each dataset, with bubble sizes representing the time complexity of all transferability scores in seconds for GT1, GT2, respectively. These values are consistent regardless of variations in ground truths. The computation times for these scores can be categorized into three groups:

- First-degree complexity: This represents a linear scale with the number of samples, denoted as *O*(*N*). Among the transferability scores, NCE is the most computationally efficient metric. It computes the negative conditional entropy between the source and target labels by evaluating probabilities for every combination of source and target labels. This involves evaluating the probabilities by checking every combination of source and target labels. While its computation time depends on the dataset size and the number of classes, NCE performs very well even with large datasets, so it is computationally efficient. Similar efficiency characteristics apply to the metrics LEEP and H-score. LEEP uses a pretrained classifier to determine probabilities, so it is computationally efficient, especially with large datasets, as its computation time primarily depends on the number of samples and classes. H-score, which computes metrics based on feature analysis and averages, is relatively fast due to its dependence only on the number of samples and features.
- Second-degree complexity: This represents a linear scale with the number of samples multiplied by the number of classes *C*, denoted as $O(C\times N)$. GBC and LogME are approximately $10 \times$ slower than NCE. However, they are still fast. GBC involves obtaining pair-class means and sample covariance, then calculating the Bhattacharyya distance between classes. It compares Gaussian distributions, so it is faster for smaller class sizes but less affected by the number of samples. LogME benefits from a carefully designed algorithm that significantly reduces computational costs. It uses a GMM to compute likelihoods. It uses a GMM to compute likelihoods. While it is slower due to considering the number of GMM components, this aspect is usually manageable, as GMM often has fewer components compared to the number of samples or features, mitigating the computational burden. EMMS uses zero-shot label embedding using the foundation model, which provides a generic and efficient computation approach. ETran, which is label and optimization-free, efficiently handles large datasets but involves computing the LDA classifier. PACTran optimizes a PAC-Bayes bound on the entire dataset, resulting in a linear scaling of running time with the number of samples, denoted as $O(C\times N)$.
- Third-degree complexity: The previous metrics demonstrate resilience to a large number of images and classes. This means these methods are relatively fast, as their performance is primarily dependent on the number of samples, outperforming methods with higher computational demands. However, TransRate, SFDA, NCTI, LFC, and $\mathscr {N}$LEEP represent the highest computation demands, particularly with larger-sized datasets. TransRate computes the coding rate of the features and their class conditional coding rates, which has a complexity of $O(N \times d)$, where *d* represents the number of features, so the running time scales linearly with the number of samples and features. Moreover, SFDA also has a complexity of $O(N \times d)$ for the ConfMix operation on the features and an additional complexity of $O(N \times d^2)$ for fitting an LDA classifier. Consequently, the total complexity of SFDA is dominated by the LDA step. NCTI performs dimensionality reduction using Principal Component Analysis (PCA) with a complexity of $O(N \times d^2)$, followed by an LDA classifier. Therefore, the running time scales quadratically with the number of samples due to the PCA and LDA steps. LFC computes covariance matrices for features and labels, resulting in a complexity of $O(N^2 \times d)$. If the number of samples exceeds 45,000, LFC randomly samples 45, 000 samples, maintaining a complexity of *O*(*N*). Thus, the running time scales quadratically with the number of samples due to the covariance matrix computation. Finally, $\mathscr {N}$LEEP performs PCA, fits the GMM to the PCA-reduced features, and computes conditional probabilities using *k* GMM components. Its total complexity is $O(N * d^2 \times k)$, which is quite high and scales linearly with the number of samples. $\mathscr {N}$LEEP ends up with an undesirably high cost because it takes a very long time to train the GMM on the target dataset, similar to the feature extraction method.

The time complexity presented here is specific to CNNs after extracting features from the datasets and then averaging them. Feature extraction is relatively inexpensive compared to brute-force methods. However, the time required for feature extraction varies significantly, ranging from about 5 min for the DTD dataset to 6 h for the Food dataset, as observed using an NVIDIA Quadro RTX 8000 GPU in this study.

Figures 8 and 9, along with 7 illustrate that LEEP, H-score, LogME, and SFDA perform well both in terms of average weighted Kendall-Tau and time complexity across most datasets, as discussed in detail earlier, while Figure S3 in the Online Appendix presents a radar chart of the top-performing scores for each dataset specifically for ViT PTMs. In addition, in the Online Appendix, Table S5provides a comprehensive summary of the overall performance by presenting the average weighted Kendall-Tau for all transferability scores across each dataset for both CNNs and ViT.

#### Evaluating scores in resource-constrained environments

Scores like TransRate, SFDA, NCTI, LFC, and $\mathscr {N}$LEEP often have high computational complexity, scaling quadratically or cubically with dataset size. This results in substantial processing time and resource requirements, particularly for large datasets. In resource-constrained environments-such as those with limited GPU availability, time constraints, or energy efficiency requirements-these high computational demands can make the use of such scores impractical or excessively costly. However, SFDA has consistently delivered strong performance across a wide range of datasets, which may justify its computational cost even in these settings. On the other hand, the more variable performance of TransRate, NCTI, and LFC raises concerns about the justification for their substantial resource requirements. Despite its computational intensity, $\mathscr {N}$LEEP has shown improvements over LEEP in certain scenarios, suggesting that it may still be a good option in certain contexts where the potential benefits outweigh the resource challenges.

However, there are situations where these scores might still be worth using, even in resource-constrained environments. First, in critical applications such as medical imaging or autonomous driving, where model selection is crucial, the additional computational cost could be justified if it leads to significantly improved outcomes. Second, in research or benchmarking settings, the deep insights provided by these computationally intensive scores can offer substantial value, forming a solid foundation for future studies or developments. Third, if transferability analysis is a rare or one-time task, the computational expense might be deemed acceptable given the potential benefits in model selection. Fourth, when simpler, faster scores fail to deliver reliable transferability estimates, employing more complex scores might be necessary. Lastly, organizations with access to substantial computational resources may find the trade-off between cost and performance acceptable, making the use of these scores more feasible.

When considering alternatives and strategies for managing the high computational demands of these scores, several approaches can be explored. One option is to use sampling strategies, applying these scores to a representative subset of the data, which can significantly reduce computational load while still providing valuable insights. A hierarchical approach can also be effective, where faster scores like LEEP and H-score are initially used to screen models, and only the most promising candidates are subjected to more computationally intensive scores. It is also important to select scores based on the specific characteristics of the dataset, which can help avoid unnecessary computations. Finally, there is a continuing need to develop more efficient scores that balance computational efficiency and predictive power, which could improve their practical application in different contexts.

## Performance variability of transferability scores across different dataset types

Different transferability scores offer varied performance depending on the characteristics of the datasets they are applied to, as demonstrated by the following examples:

1. NCE with CNN-based models may outperform ViT-based models on texture datasets like DTD. However, NCE performs poorly on datasets requiring global features or detailed object understanding. This is due to several key reasons. NCE operates under the assumption that the input data for both tasks is the same, with differences only in label assignments. Consequently, NCE measures the compatibility between source and target label distributions without relying on the specific features extracted by the model. For texture datasets, where classes are defined by visual patterns rather than distinct object categories, this label-distribution-based approach is particularly effective. On the other hand, CNN architectures excel with texture datasets due to their ability to capture local features. CNN-based features align well with texture characteristics because lower-level CNN features, such as edge detectors and color blobs, are particularly suited for describing textures. Convolution filters in CNNs focus on small, localized regions of an image, which is ideal for recognizing repeating patterns and textures. In contrast, ViT-based scores tend to perform less effectively on texture datasets because they focus on global image structures, which are less relevant for textures that depend on local patterns. Furthermore, ViTs lack the spatial inductive bias found in CNNs, making them less suited for tasks where local spatial relationships are crucial.
2. LEEP performs well with large-scale coarse-grained datasets where empirical distributions are stable, leading to accurate transferability assessments. Conversely, LEEP’s performance declines on fine-grained datasets. Several factors influence the performance variation of these scores: LEEP uses empirical distributions and pseudo-labels from PTMs, which work well with large datasets, providing accurate transferability assessments. For these datasets, the richer data and more reliable pseudo-labels lead to robust estimates. However, LEEP struggles with smaller or fine-grained datasets like Flowers or Cars because the pretrained model may not have seen similar categories, resulting in less accurate pseudo-labels and unreliable scores.
3. $\mathscr {N}$LEEP is an advanced version of LEEP, that includes a GMM to better estimate the density of pseudo-labels. This approach is particularly effective for large datasets with clear class separations, such as Cifar10. However, $\mathscr {N}$LEEP struggles with datasets characterized by high-dimensional feature spaces or overlapping classes and fine-grained datasets. In these complex scenarios, the GMM may fail to capture fine distinctions, leading to less accurate assessments of transferability.
4. GBC works well for datasets with a limited number of distinct classes and lower-dimensional features like Cifar10. It evaluates class separability using the Bhattacharyya distance, which works well when class distributions are separable. However, the distance measurements can become unstable and the performance degrades on datasets with many overlapping class distributions and the difficulty of accurately estimating covariance matrices, leading to less reliable assessments of class separability and transferability.
5. SFDA performs well on larger datasets like Cifar10 and Cifar100, where its ability to finetune and handle diverse feature combinations significantly improves class separability. The method’s effectiveness is due to its ConfMix operation, which leverages class-conditional probabilities to enhance feature and separation. However, SFDA faces difficulties with smaller, fine-grained datasets. In these cases, the limited amount of data restricts the effectiveness of feature mixing, and the complex class structures pose challenges for SFDA and lead to less effective class separation and reduced transferability.
6. Etran works effectively when there is a clear distinction between source and target distributions. It accurately classifies datasets as IND or OOD when differences are significant. However, it faces challenges with small, fine-grained datasets where minor class differences complicate the assessment of distribution alignment, leading to unreliable transferability scores. Additionally, Etran’s reliance on LDA for classification further magnifies its challenges with fine-grained datasets. LDA can be prone to overfitting in scenarios where there are many visually similar categories. This overfitting can lead to inaccuracies in class separation and, consequently, unreliable transferability estimates.
7. EMMS leverages foundation models’ capabilities to evaluate and select PTMs for various tasks. It performs well on datasets with broad, general categories that align with the knowledge embedded in these foundation models. However, EMMS faces challenges with specialized datasets that demand a complex understanding of fine-grained details or specific domains, such as texture or small-scale fine-grained datasets.

These examples emphasize that understanding the specific dataset characteristics is key to choosing an effective transferability score, which demonstrates that no single score is universally optimal across all scenarios.

## Guidelines for choosing transferability scores in image classification

To summarize the findings and provide general recommendations, the following guidelines are proposed for selecting the appropriate transferability scores in image classification tasks, considering dataset characteristics and problem-specific requirements:

- For fine-grained datasets that require analysis of subtle differences, such as distinguishing between similar species or object types (e.g., Aircrafts, Flowers, and Cars datasets), information theory-based scores like LEEP and $\mathscr {N}$LEEP might be prone to overfitting on limited data and are generally not recommended. Instead, feature space analysis (e.g., SFDA) or Bayesian approaches (e.g., LogME) may be more appropriate options. However, their effectiveness can vary depending on class separability and the complexity of class distributions. The performance of EMMS may also be sub-optimal unless the underlying foundation models have been pretrained on similar domains.
- For large-scale fine-grained datasets like classifying animals into broad categories, information theory-based scores may perform poorly in the presence of noisy pseudo-labels. Bayesian scores like LogME might also struggle when dealing with complex and overlapping class distributions.
- For texture datasets, such as DTD, NCE performs well due to its underlying assumptions that align with the characteristics of texture data. In addition, CNN-based scores tend to outperform ViT-based scores in capturing textural details.
- For the case of simple coarse-grained datasets like Cifar10, Cifar100, and VOC2007, most scores show satisfactory performance, with feature space analysis and Bayesian scores being particularly effective. ViT-based scores also demonstrate strong performance, likely due to improved class separability.
- For datasets with a limited number of classes, the H-score is beneficial due to its focus on class separation and generalization ability.
- If computational efficiency is a priority, LEEP and H-score are recommended for their low computational overhead. Conversely, it is advisable to avoid using scores like TransRate, SFDA, NCTI, LFC, and $\mathscr {N}$LEEP for large-scale datasets due to their significant computational demands.
- When using ViT PTMs, most transferability scores generally perform better compared to CNN models. These scores are particularly effective for fine-grained datasets, probably due to ViT’s ability to learn global features. However, scores such as LEEP and LogME are recommended in this case, as they leverage the unique characteristics of transformer architectures and their handling of contextual information. On the other hand, when using CNNs, scores like TransRate and GBC can effectively evaluate transferability due to their focus on feature representation and mutual information.
- Further recommendations for future research include the potential utility of ETran in scenarios involving OOD data or domain shifts, despite its possible sensitivity to variations in ground truth. Similarly, EMMS requires investigation for multi-task or multi-modal problem domains due to its potential versatility across various tasks and modalities. In addition, LFC and NCTI may be promising in contexts characterized by limited data or few-shot learning, although their effectiveness in these constrained conditions has yet to be empirically validated.

**Fig. 10:**
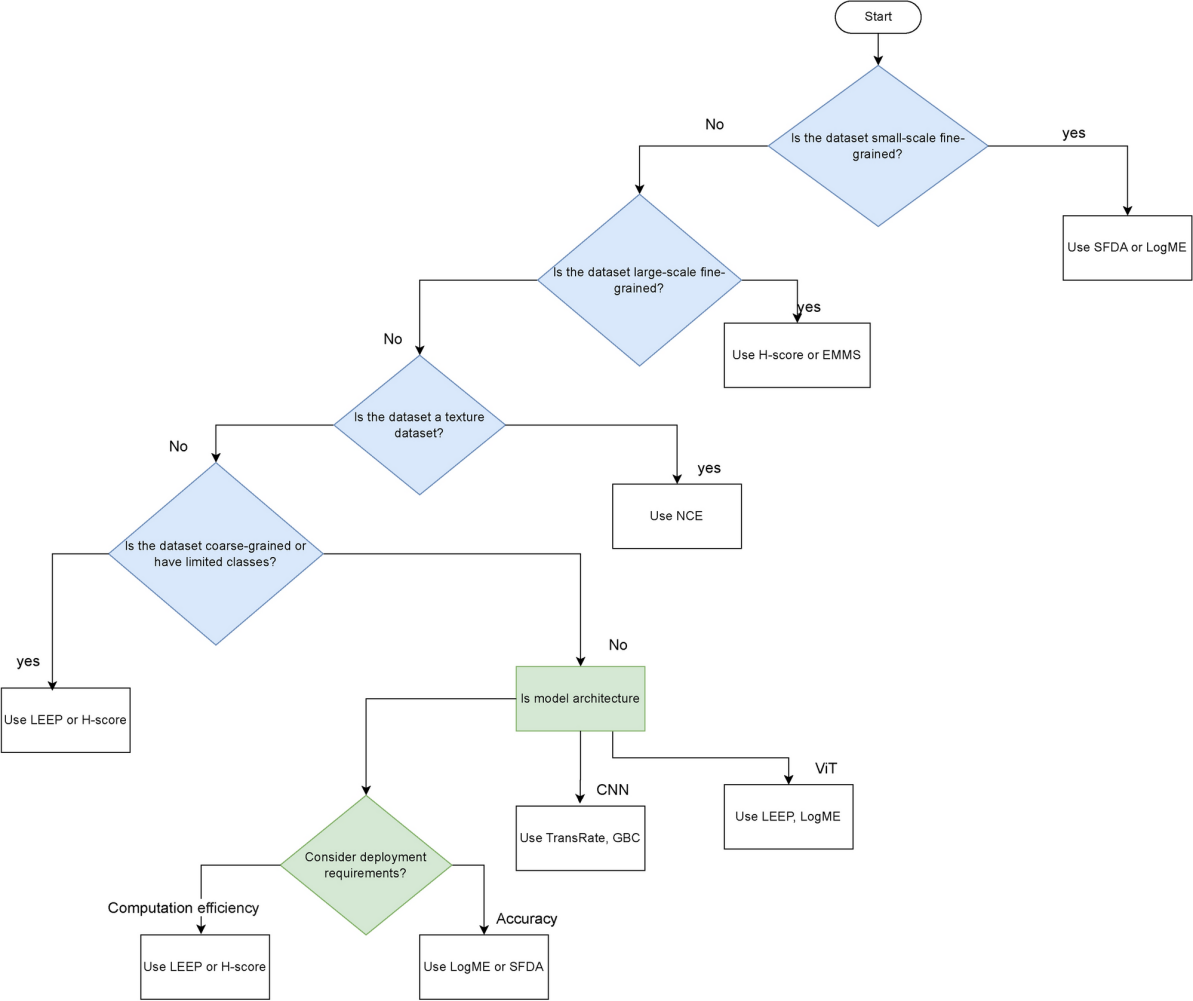
Decision tree for selecting transferability scores based on dataset characteristics and constraints.

However, the study demonstrates the absence of a universally superior score across all scenarios. Practitioners are recommended to carefully consider the specific characteristics of their dataset, such as its size, class count, and domain, as well as their computational resources and the nature of the task when selecting a transferability score. Moreover, employing multiple scores, when possible, is recommended to ensure a more comprehensive evaluation of transferability. To help practitioners address these considerations, Fig. 10 visualizes the decision tree. It summarizes the process for selecting transferability scores based on dataset characteristics and constraints to better understand the landscape of existing research.

To provide practical suggestions with specific examples of transferability scores, the following strategy is recommended for practitioners:

1. Start with a baseline score: Begin with LEEP or H-score due to their balance of accuracy and efficiency. These scores provide a reliable baseline for transferability assessment, as they are computationally efficient and tend to perform well across a range of datasets and tasks.
2. Adjust based on results: If higher accuracy is desired and computational resources allow, consider integrating LogME or SFDA. Combining scores like LogME with SFDA can provide a more comprehensive evaluation of model transferability, as SFDA captures finetuning dynamics while LogME provides robust generalization.
3. Consider dataset and problem constraints: Choose scores based on the specific features of the target dataset and the limits of the problem. For instance, if working with fine-grained classification datasets, $\mathscr {N}$LEEP or PACTran might be better suited as they are designed to handle more complex class separability. For large and diverse datasets, sticking with LogME or SFDA could yield better generalization across tasks. More details about the dataset types with the performance of transferability scores are shown in Table 2.

This strategy leverages the strengths of various transferability scores, allowing practitioners to adapt their model selection process to the specific needs of their projects.

## Conclusion

This study provides a comprehensive evaluation of various transferability scores used for selecting PTMs in image classification tasks. The findings indicate that the performance of these scores is influenced by several factors, including dataset size, number of classes, class granularity, domain shift, feature space complexity, and the adaptation of PTMs to the target domain or task. The results demonstrate the varying degrees of success and limitations of the evaluated scores across various dataset types. For instance, scores like LEEP, H-score, LogME, and SFDA generally demonstrate superior performance in multiple cases, but their effectiveness is context-dependent and requires careful selection based on specific datasets and task requirements. The study demonstrates the importance of robust transferability scores that can generalize well to OOD datasets, fine-grained datasets, and high intra-class variation. Additionally, computational efficiency emerges as a crucial consideration, particularly in resource-constrained environments. This work thus offers empirical insights and practical guidance for practitioners in selecting and utilizing transferability metrics for image classification tasks. It emphasizes the need for a superior transferability metric that balances accuracy, computational efficiency, and dataset characteristics.

Looking ahead, several key issues remain unresolved, suggesting promising avenues for future research. One direction is to explore the applicability of transferability scores in diverse domains, such as medical imaging and NLP, to assess their generalizability. Another area of interest is investigating the performance of these scores in low-data scenarios, such as few-shot learning, which could offer valuable insights for practitioners working with limited annotated data. Additionally, further research is needed to examine the interaction between transferability scores and domain adaptation techniques. Understanding how these scores perform when models are finetuned using different adaptation methods could lead to more robust transfer learning strategies. Furthermore, despite the effectiveness of current scores, there is potential to develop new metrics that address the limitations of existing approaches, especially in balancing accuracy with computational efficiency for large-scale or real-time applications. Future research should focus on varying experimental parameters to create transferability scores that are both robust and adaptable to different dataset characteristics. This approach will enhance the practical utility of these scores, improving model selection and transfer learning practices.

## Supplementary Information

Supplementary Information.

## Data Availability

https://github.com/NanoAB86/ModelSelection_Benchmark.

## Code availability

The code is available on Github: https://github.com/NanoAB86/ModelSelection_Benchmark.
