# Supplementary material for: One size does not fit all in evaluating model selection scores for image classification
Source: Sci Rep. 2024 Dec 4;14:30239. doi: 10.1038/s41598-024-81752-w (PMC11618499; doi:10.1038/s41598-024-81752-w)
Supplement: Supplementary file 1 — Supplementary Information. [file 41598_2024_81752_MOESM1_ESM.pdf]

Appendix

Datasets overview

The table S1 lists the number of images and classes for each tested dataset to provide context on the dataset characteristics.

Datasets	Number of images	Number of classes	Task type
DTD	5,640	47	Texture classification
Oxford IIIT Pets	7,349	37	Fine-grained classification
Oxford-102 Flowers	8,189	102	Fine-grained classification
Caltech101	9,144	101	Coarse-grained classification
VOC2007	9,963	20	Coarse-grained classification
FGVC Aircraft	10,000	100	Fine-grained classification
Stanford Cars	16,185	196	Fine-grained classification
SUN397	39,700	397	Scene classification
Cifar10	60,000	10	Coarse-grained classification
Cifar100	60,000	100	Coarse-grained classification
Food101	101,000	101	Fine-grained classification

Table S1. The number of images, classes, and task types for the tested datasets

Ground truths values and sensitivity analysis

Tables S2, S3 present the ground truths accuracy values obtained by the pretrained models on the target datasets, as a reference point for evaluating the effectiveness of the transferability scores in predicting these performance levels. Then, the figure S1 shows the absolute percentage change in total scores between two sets of ground truths values, resulting in the sensitivity of certain metrics to variations in ground truths labels. Table S4 shows the ViT GT. In each table, the highest accuracy achieved for every dataset is indicated in bold font.

Dataset	Models									
	Res50	Res101	Res152	Dense121	Dense169	Dense201	MnasNet1	MobileNetV2	GoogleNet	InceptionV3
Aircrafts	86.6	85.6	85.3	85.4	84.5	84.6	72.8	82.8	82.7	**88.8**
Caltech101	91.8	93.1	93.2	91.9	92.5	93.4	91.5	89.1	91.7	**94.3**
Cars	91.7	91.7	92.0	91.5	91.5	91.0	88.5	91.0	91.0	**92.3**
CIFAR10	96.77	**97.68**	97.91	97.18	97.4	97.41	96.83	95.74	96.17	97.5
CIFAR100	84.5	87.0	**87.6**	84.8	85.0	86.0	83.9	80.8	83.2	86.6
DTD	75.2	76.2	75.4	74.9	74.8	74.5	72.8	72.9	73.6	**77.2**
Flowers	97.51	97.94	97.35	97.18	97.86	97.68	96.83	96.63	97.26	**97.98**
Food	87.8	87.6	87.6	87.7	88.0	87.3	85.5	86.2	85.6	**88.8**
Pets	92.5	94.0	**94.5**	92.9	93.1	92.8	89.4	90.5	91.9	93.5
Sun397	64.7	64.8	**66.0**	62.3	63.0	64.7	60.7	60.5	62.0	65.7
VOC2007	85.7	**86.6**	**86.6**	85.1	85.9	85.8	83.5	82.1	83.2	**86.6**

Table S2. Ground truths GT1

Dataset	Models										
	Res34	Res50	Res101	Res152	Dense121	Dense169	Dense201	MnasNet1	MobileNetV2	GoogleNet	InceptionV3
Aircrafts	84.06	84.64	85.53	**86.29**	84.66	84.19	85.38	66.48	79.68	80.32	80.15
Caltech101	91.15	91.98	92.38	93.1	91.5	92.51	**93.14**	89.34	88.64	90.85	92.75
Cars	88.63	89.09	89.47	**89.88**	89.34	89.02	89.44	72.58	86.44	87.76	87.74
CIFAR10	96.12	96.28	97.39	**97.53**	96.45	96.77	97.02	92.59	94.74	95.54	96.18
CIFAR100	81.94	82.8	84.88	**85.66**	82.75	84.26	84.88	72.04	78.11	79.84	81.49
DTD	72.96	74.72	74.8	**76.44**	74.18	74.72	76.04	70.12	71.72	72.53	72.85
Flowers	95.2	96.26	96.53	96.86	97.02	**97.32**	97.1	95.39	96.2	95.76	95.73
Food	81.99	84.45	85.58	86.28	84.99	85.84	**86.71**	71.35	81.12	79.3	81.76
Pets	93.5	93.88	93.92	**94.42**	93.07	93.62	94.03	91.08	91.28	91.38	92.14
Sun397	61.02	63.54	63.76	**64.82**	63.26	64.1	64.57	56.56	60.29	59.89	59.98
VOC2007	84.6	85.8	85.68	**86.32**	85.28	85.77	85.67	81.06	82.8	82.58	83.84

Table S3. Ground truths GT2

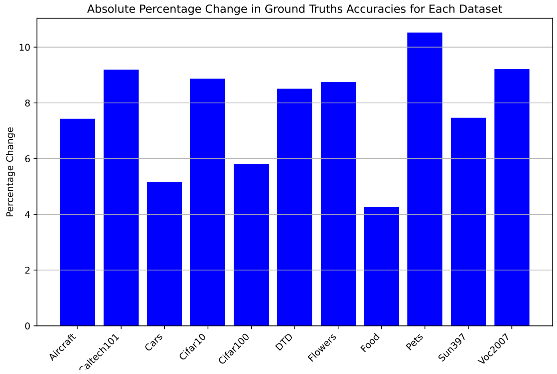

Figure S1. The absolute percentage change of the total scores for each dataset between the two sets of ground truths scores

Dataset	Models												
	DeiT_Tiny	DeiT_Small	DeiT_Base	DINO_Small	DINO_Base	MoCoV3_Small	PVTv2_B2	PVTv2_B3	PVT_Tiny	PVT_Small	PVT_Medium	Swin_T	Swin_S
Aircrafts	71.26	73.12	78.39	72.18	67.13	76.04	84.14	**84.7**	69.76	75.2	76.7	81.9	83.24
Caltech101	89.39	92.7	93.47	86.76	92.34	89.84	93.13	**94.4**	90.04	93.02	93.75	91.9	94.0
Cars	82.09	86.72	89.26	79.81	80.74	82.18	90.6	**91.22**	84.1	87.61	87.66	88.93	89.81
CIFAR10	96.52	97.69	**98.56**	97.96	98.31	97.92	97.96	98.44	94.87	97.34	97.93	97.34	98.06
CIFAR100	81.58	86.62	**89.96**	85.66	89.38	85.84	88.24	89.3	75.26	86.2	87.36	85.97	88.42
DTD	71.86	75.08	**77.66**	75.96	76.01	71.88	77.16	77.37	72.92	75.77	77.1	77.04	77.34
Flowers	95.5	96.79	97.98	95.96	96.28	93.89	97.89	**98.06**	95.8	97.32	97.36	97.4	96.87
Food	81.96	86.26	88.96	85.69	87.1	82.84	88.67	**89.08**	83.78	86.98	85.56	86.67	87.7
Pets	91.44	94.02	94.61	92.59	93.41	90.44	93.86	**95.14**	91.48	94.13	94.48	94.5	94.8
Sun397	58.4	64.76	**68.62**	64.14	64.78	60.6	66.44	67.54	61.86	65.78	67.22	65.51	67.03
VOC2007	83.1	86.62	87.88	84.8	86.72	81.84	86.44	88.08	84.6	86.62	87.36	87.54	**88.26**

Table S4. ViT GT

The pseudo-code of the proposed framework

The following algorithm S1 represents the pseudo-code of the proposed framework of the model selection process.

Algorithm S1 Transferability Evaluation Framework

Input: Target dataset D_t, pretrained models pool $\mathscr{M}$
Output: Selected model $\mathscr{M}^*$
Step 1: Collect Pretrained Models
Collect a pool of pretrained models $\mathscr{M}$, including CNN and ViT models, pretrained on large datasets such as ImageNet
Step 2: Extract Features
for each model $m_i \in \mathscr{M}$ **do**
  Pass target dataset D_t through m_i
  Extract activations from the penultimate layer of m_i
  Store extracted features F_i for m_i
end for
Step 3: Evaluate Transferability Scores
for each model $m_i \in \mathscr{M}$ **do**
  For each transferability score:
  for each score type **do**
    `tic` {Start timing}
    Calculate the transferability score using extracted features F_i and target dataset labels
    `toc` {End timing}
    Record the computational time for this score
  end for
end for
Step 4: Computational Efficiency Analysis
for each model $m_i \in \mathscr{M}$ **do**
  Compute the average computational cost across all transferability scores for m_i
  Store the average computational cost for m_i
end for
Step 5: Rank Models
Rank models $\mathscr{M}$ based on transferability scores and average computational cost
Higher scores and lower costs indicate better performance and efficiency
Step 6: Model Selection
Select the pretrained model $\mathscr{M}^*$ that provides the best trade-off between the highest transferability score and average computational cost
Step 7: Output
Output the selected model $\mathscr{M}^*$

Comparison of Weighted Kendall-Tau by Dataset & Model

The table S5 presents the average Weighted Kendall-Tau for all transferability scores for each dataset for CNNs and ViT, summarizing the overall performance.

Averages	DTD	Pets	Flowers	Caltech101	VOC2007	Aircraft	Cars	SUN397	Cifar10	Cifar100	Food
GT1	0.26	0.50	0.18	0.58	0.63	0.07	0.29	0.60	0.74	0.73	0.11
GT2	0.55	0.45	0.13	0.53	0.52	0.03	0.35	0.52	0.76	0.69	0.48
ViT	0.28	**0.49**	**0.27**	0.28	0.39	**0.16**	**0.41**	0.39	0.53	0.49	**0.38**

Table S5. Average weighted Kendall-Tau for transferability scores by dataset and model architecture

Progression of top-performing transferability scores across datasets

Figure S2 shows the radar charts of the top-performing transferability scores for each dataset, providing an at-a-glance view of the relative strengths and weaknesses of different metrics across various data characteristics for CNN pretrained models on

GT1 and GT2. Additionally, figure S3 shows the radar chart corresponding to the ViT pretrained models. The figures show that the best-performing score for one dataset can be very different from others, indicating that each dataset and model setup has its own unique characteristics influencing performance.

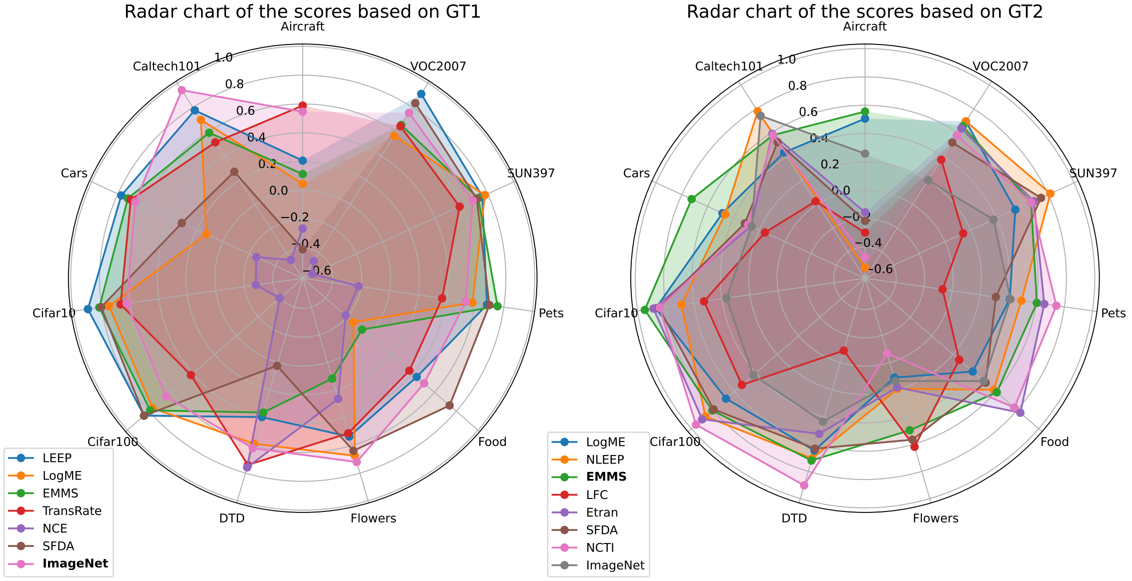

Figure S2. the radar chart for the top-performing scores only for each dataset for CNN pretrained models

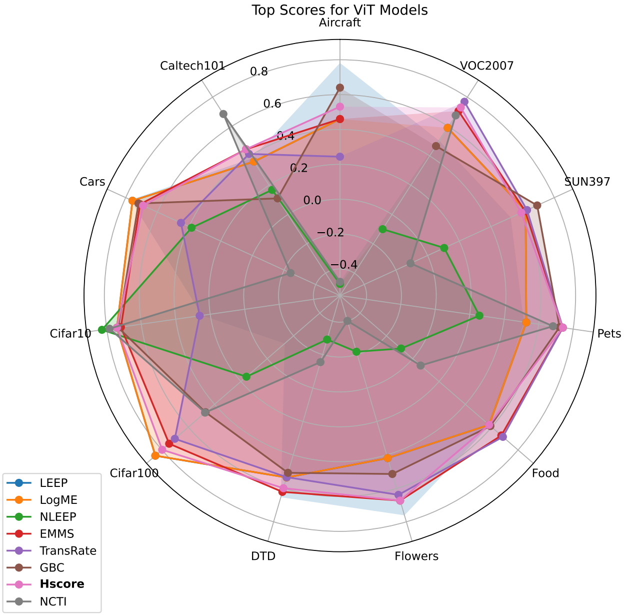

Figure S3. the radar chart for the top-performing scores only for each dataset for ViT pretrained models
